# Molecular mechanism differences between nanoplastics and microplastics in colon toxicity: nanoplastics induce ferroptosis-mediated immunogenic cell death, while microplastics cause cell metabolic reprogramming

**DOI:** 10.1186/s12951-025-03545-1

**Published:** 2025-07-14

**Authors:** Yixian Cheng, Junjie Chen, Rui Fu, Peng Zhang, Haosong Chen, Haikun Cao, Zilong Jiang, Yuan Hong, Yifan Li, Cuiqi He, Fengjie Tao, Ting Li, Jiawei Zhang, Bo Chen, Guodong Cao

**Affiliations:** 1 Department of General Surgery, The First Affiliated Hospital of Anhui Medical University, Hefei, Anhui China; 2 Department of Surgical Oncology, The First Affiliated Hospital of Bengbu Medical University, Bengbu, China; 3 Department of Medical Oncology, The First Affiliated Hospital of Anhui Medical University, Hefei, China

**Keywords:** Nanoplastics, Microplastics, Ferroptosis, Fosl1, Immunogenic cell death, YAP signaling, Metabolic reprogramming

## Abstract

This study investigates the size-dependent mechanisms by which polystyrene (PS) microplastics (MPs) cause intestinal epithelial injury, focusing on the differential effects of nanoscale (100 nm) and microscale (10 μm) particles. Using both cellular and animal models, we found that 100 nm MPs are internalized by intestinal epithelial cells via endocytosis, triggering oxidative stress and ferroptosis, while 10 μm MPs primarily induce epithelial damage through mechanical disruption and metabolic reprogramming. Proteomic analyses revealed that small MPs significantly upregulate ferroptosis-related genes, including Fosl1 and components of the p53 pathway. Functional studies showed that Fosl1 promotes p53 transcription, suppresses Slc7a11, and contributes to ferroptosis. Mechanistically, ferroptosis was characterized by intracellular iron accumulation, glutathione (GSH) depletion, GPX4 inactivation, and excessive lipid peroxidation. Treatment with Ferrostatin-1 or Fosl1 knockdown alleviated ferroptosis and epithelial injury. In contrast, large MPs activated the mechanosensitive YAP pathway, leading to cytoskeletal remodeling and a metabolic shift from oxidative phosphorylation to anaerobic glycolysis. This metabolic reprogramming was associated with increased inflammation. These findings demonstrate that microplastic particle size critically determines toxicity mechanisms—ferroptosis for small particles and YAP-mediated metabolic disruption for large ones—and suggest potential therapeutic targets for mitigating microplastic-induced intestinal damage.

**Graphical abstract:**

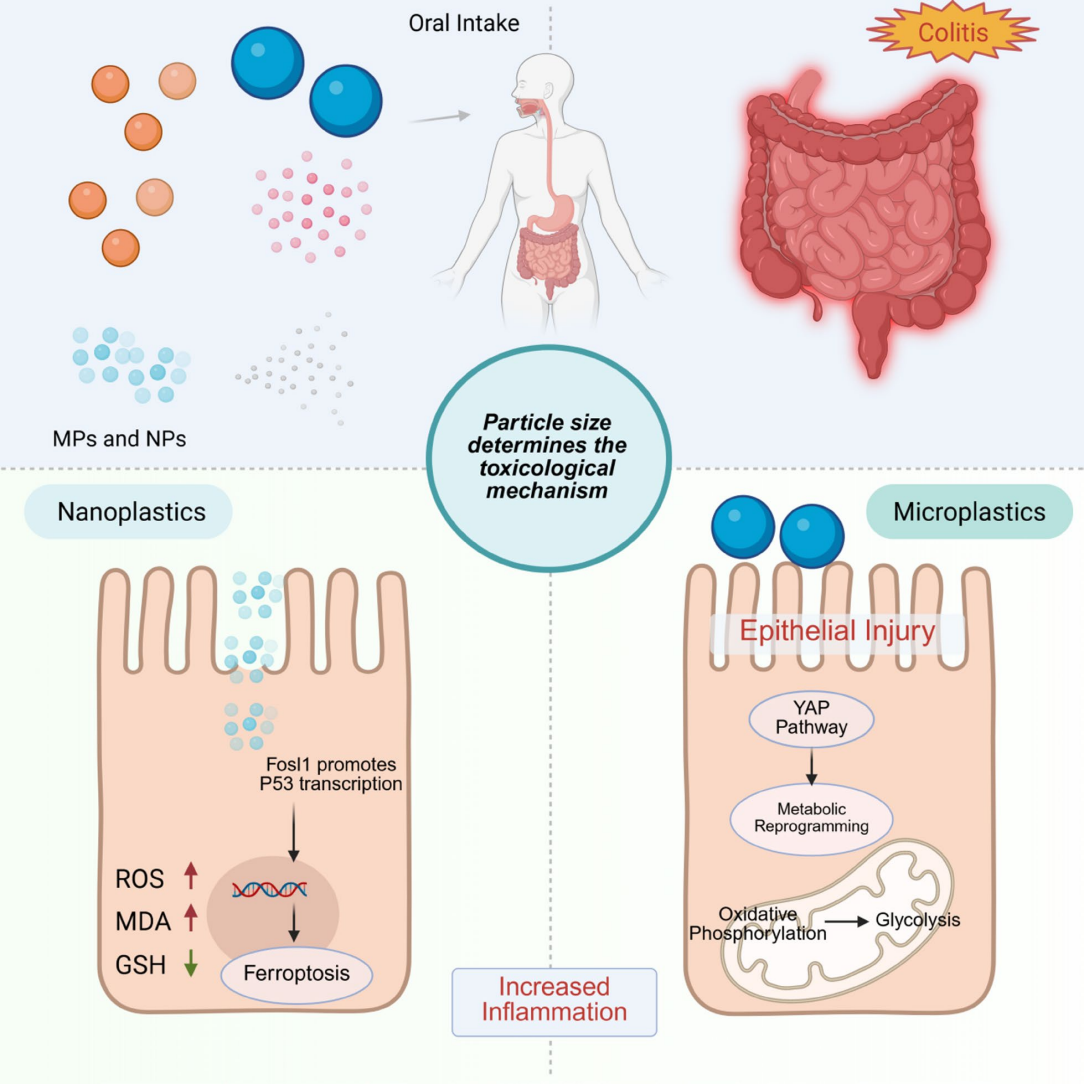

**Supplementary Information:**

The online version contains supplementary material available at 10.1186/s12951-025-03545-1.

## Introduction

The increasing accumulation of plastic waste has become a significant global environmental issue, drawing widespread concern. As an inexpensive and highly versatile synthetic material, plastic has found widespread application across numerous sectors, including packaging, household goods, construction materials, and medical devices. The remarkably slow decomposition of plastic results in its long-term persistence in natural ecosystems, thereby intensifying environmental concerns. Currently, over 400 million tons of plastic are produced annually [1], resulting in the accumulation of vast quantities of plastic waste that cause significant harm to ecosystems and biodiversity [2, 3].

In recent years, microplastics (MPs) and nanoplastics (NPs), which arise from the degradation or fragmentation of larger plastic debris, have been increasingly detected in diverse environmental matrices, including oceans, freshwater systems, soil, air, and even the food chain. These particles can be ingested by marine organisms and terrestrial animals, bioaccumulate through trophic transfer, and ultimately enter the human body via seafood, drinking water, and agricultural products [4].

Microplastics and nanoplastic particles are degradation by-products of plastic, which are ubiquitously distributed in the environment and have the potential to enter organisms via the food chain. The most common microplastics in the environment are polyethylene (PE), polyvinyl chloride (PVC), polypropylene (PP), polyethylene Terephthalate (PET) and polystyrene (PS) [5]. Microplastics generally refer to plastic particles smaller than 5 mm [6, 7]. While a universal definition for nanoplastics (NPs) remains elusive, most studies describe them as plastic particles ranging from 1 to 1000 nanometers in size [2, 8, 9].

Recent research has shifted from a purely environmental perspective to growing concern about the potential impacts of MPs and NPs on biological systems. Owing to their small size and physicochemical characteristics, these particles are capable of interacting with cells and tissues, thus posing risks to biological health [10, 11]. MPs and NPs are primarily ingested through the oral route and tend to accumulate in the gastrointestinal (GI) tract over time. Once in the intestine, they directly interact with intestinal epithelial cells, compromising epithelial barrier integrity, disrupting immune balance, and affecting gut health. These disruptions can trigger inflammatory responses, damage mucosal structures, and potentially promote tumorigenesis, contributing to a cascade of gastrointestinal disorders [12–15].

As the first point of contact for ingested MPs and NPs, intestinal epithelial cells play a vital role in maintaining gut barrier function and immune homeostasis [16–18]. Consequently, significant attention has been directed toward understanding the relationship between gut health and microplastic pollution. The intestinal barrier not only acts to prevent the invasion of harmful substances but also plays a crucial role in maintaining normal immune function [18]. The intestinal barrier prevents the translocation of harmful substances while also orchestrating immune responses. Due to their larger size, MPs generally cannot enter cells via endocytosis. Instead, they are thought to damage epithelial integrity by direct physical compression, inducing membrane rupture and triggering mechanical stress [19]. Therefore, the damage caused by MPs and NPs is likely mediated primarily through physical interactions with intestinal epithelial cells. Such contact may compromise the intestinal barrier through multiple mechanisms, including mechanical disruption, activation of immune responses, and induction of oxidative stress [14]. In contrast, due to their smaller size and larger surface area, nanoplastics are more readily absorbed by the body and can enter cells [20], triggering toxic reactions such as oxidative stress, DNA damage, and cell apoptosis [21]. Notably, nanoplastics can enter cells directly via endocytosis by intestinal epithelial cells, activating intracellular oxidative stress responses and causing cellular damage [22–26]. Furthermore, nanoplastic particles may alter the gut microbiome, further disrupting intestinal immune function and metabolic stability [27–29].

Once internalized, MPs and NPs may undergo limited biodegradation and are thought to persist in tissues, especially the liver, intestines, and lymphoid organs. The intracellular handling of NPs may involve autophagic or lysosomal pathways, while MPs may be retained within the gut lumen or taken up by macrophages [30]. However, the metabolic fate and clearance mechanisms of these particles remain poorly characterized, necessitating further investigation.

Notably, micro/nanomaterial-mediated toxicity has emerged as a critical toxicological concept. These materials can induce organ-specific dysfunction, inflammatory responses, and even systemic immune alterations depending on their size, shape, and surface properties. Such findings underscore the need to understand how nanoscale and microscale plastics exert differential biological effects [31].

Despite the expanding literature on microplastic-induced gut toxicity, few studies have systematically compared how particle size influences specific mechanisms of intestinal damage. Understanding the size-dependent toxicological pathways of MPs and NPs is critical for accurate risk assessment and the identification of targeted intervention strategies. This study addresses this gap by directly comparing PS particles across a size gradient (100 nm to 10 μm), which allows for a controlled evaluation of cellular and molecular responses to different plastic scales. Emphasizing this comparative size-based approach is a novel aspect of our work, as most previous studies have focused on a single plastic type or size. Using both in vivo (mouse) and in vitro (intestinal epithelial cell) models, we administered particles of 100 nm, 500 nm, 1 μm, 5 μm, and 10 μm via oral gavage for a 6-week period. We employed cell viability assays, transmission electron microscopy, flow cytometry, and proteomics to investigate the resulting inflammatory and molecular changes.The results indicated that 100 nm nanoplastics induced ferroptosis [32, 33]in intestinal epithelial cells. Following ferroptosis, cellular antigens were released and subsequently recognized by dendritic cells (DCs) [34, 35], which presented the antigens to CD8 + T cells [36], leading to an increased infiltration of both dendritic cells and CD8 + T cells into the intestinal mucosa. This exacerbated inflammation and further promoted gut injury. Proteomic sequencing revealed that Fosl1 expression was upregulated, suggesting its role in regulating P53 [37] transcription and participating in the ferroptosis process. The ferroptosis inhibitor Ferrostatin-1 [38, 39] was used to detect ROS, MDA and Western blot on cell experiments, and in vivo mice experiments, to further validate the occurrence and effect of ferroptosis. In contrast, the damage induced by larger microplastic particles appears to be associated with metabolic reprogramming. Proteomics and non-targeted energy metabolomics analyses suggested that glycolysis plays a role in this process. Additionally, larger microplastic particles may induce mechanical changes at the cellular level, such as compression-induced injury, which upregulates the key protein YAP [40, 41] and drives a shift in metabolism from oxidative phosphorylation to anaerobic glycolysis. Intervention with the YAP inhibitor Verteporfin [42] demonstrated that suppressing YAP translocation to the cell nucleus could potentially reverse inflammation and alleviate gut damage induced by microplastics.

This study has elucidated the potential mechanisms underlying gut inflammation induced by microplastics of different sizes, highlighting that microplastics and nanoplastics exert distinct, size-specific mechanisms that contribute to gut damage. These findings offer important theoretical insights into the molecular mechanisms underlying microplastic-induced intestinal injury, and propose potential therapeutic targets for the prevention and mitigation of such damage.

## Materials and methods

### Materials

Two types of polystyrene (PS) microplastics (Jiangsu Zhichuan, China) were used in this study. Conventional PS particles with sizes of 100 nm, 500 nm, 1 μm, 5 μm, and 10 μm were applied in animal and cell-based toxicological studies. Red fluorescent PS microplastics (Ex = 520 nm, Em = 580 nm) of the same sizes were used for visualizing uptake, accumulation, and distribution in vivo and in vitro. Particle morphology and size were confirmed by scanning electron microscopy (SEM) and laser particle size analysis. Chemical composition was validated using Fourier-transform infrared spectroscopy (FTIR).

### Animal experimental design

Four-week-old male BALB/c mice (SPF grade) were obtained from the Anhui Experimental Animal Center (Hefei, China) and housed under standardized conditions (temperature: 22–26 °C; 12-hour light/dark cycle). After a one-week acclimatization period, mice were randomly assigned into experimental groups based on the study objectives, with 5 mice per group as follows:

#### Size-dependent toxicity evaluation (*n* = 30, 5 per group)

Control (PBS), and five PS microplastic exposure groups (100 nm, 500 nm, 1 μm, 5 μm, 10 μm). Each treatment group received 200 µL of 1 mg/mL PS microplastics (equivalent to 0.1 mg/day) by oral gavage daily for 7 weeks; the control group received an equal volume of PBS.

#### Ferroptosis Inhibition experiment (*n* = 25, 5 per group)

Control, 100 nm MPs, 10 μm MPs, 100 nm MPs + Ferrostatin-1, and 10 μm MPs + Ferrostatin-1. Ferrostatin-1 (1 mg/kg) was administered via intraperitoneal injection every other day for 7 weeks, concurrent with MP gavage.

#### YAP Inhibition experiment (*n* = 15, 5 per group)

Control, 10 μm MPs, and 10 μm MPs + VTPF. VTPF (5 mg/kg) was intraperitoneally injected twice per week for 7 weeks, along with daily gavage of 10 μm MPs (0.1 mg/day).

Across all experiments, prior to the final gavage, mice were weighed and fasted for 8 h. They were then euthanized via cervical dislocation. Blood samples and gastrointestinal tissues—including the stomach, cecum, and colon—were collected for subsequent histological, biochemical, and immunological analyses. All animal protocols were reviewed and approved by the Animal Ethics Committee of Anhui Medical University and adhered to institutional guidelines for animal welfare.

### Cell culture and SiRNA transfection

The human colonic epithelial cell line NCM460 was obtained from Wuhan Warner Biological Technology. Cells were maintained in RPMI 1640 medium (Gibco, USA) supplemented with 10% fetal bovine serum, 100 IU/mL penicillin, and 100 µg/mL streptomycin at 37 °C in a humidified 5% CO₂ incubator. Upon reaching > 90% confluence, cells were passaged and seeded into 6-, 24-, or 96-well plates for subsequent experiments. Fosl1-specific siRNAs and YAP-specific siRNAs were transiently transfected into NCM460 cells using the jetPRIME transfection reagent, following the manufacturer’s instructions. The siRNA oligonucleotide sequences were as follows: Fosl1-siRNA-1: 5′-GCUCAUCGCAAGAGUAGCA-3′ and Fosl1-siRNA-2: 5′-GAGCUGCAGUGGAUGGUAC-3′ and YAP-siRNA-1: 5′-GCCATTAAAGGCAGCTGTTC-3′ and YAP-siRNA-2: 5′-AGCACTGTGCCAGGTATCAC-3′. Briefly, siRNAs were diluted in jetPRIME buffer, mixed with the transfection reagent, and incubated at room temperature for 10 min before being added to the cells. After 12 h of transfection, the medium was replaced with fresh complete culture medium, and the cells were incubated for an additional 48 h. Cells were then harvested for protein extraction, and the knockdown efficiency of Fosl1 and YAP was confirmed by Western blot analysis.

### Characterization of polystyrene microplastics

Polystyrene (PS) microplastic particles of five sizes (100 nm, 500 nm, 1 μm, 5 μm, and 10 μm) were purchased from ZhiChuan Technology (Jiangsu, China). The morphology and dispersion of particles were examined using scanning SEM at both low and high magnifications. Particle size distributions were analyzed with a laser particle size analyzer. The chemical composition of all PS microplastics was confirmed using FTIR.

### Uptake of fluorescently labeled PS microplastics of different sizes by normal Colon epithelial cells

NCM460 cells were seeded into 24-well plates and cultured for 24 h to ensure stable growth. Red fluorescent polystyrene (PS) microplastics of various sizes were added at 1 mg/mL and co-incubated with the cells for another 24 h. After incubation, wells were rinsed with PBS to remove non-internalized particles. Red fluorescence and DAPI-stained nuclei were visualized using an Olympus inverted fluorescence microscope.

### Histopathological staining of tissues

Colon inflammation was evaluated by histopathological analysis. Tissues were fixed in 4% paraformaldehyde, dehydrated with graded ethanol, embedded in paraffin, and sectioned at 4 μm thickness. Hematoxylin and eosin (H&E) and Alcian Blue–Periodic Acid–Schiff (AB-PAS) staining were performed using commercial kits (Solarbio, Beijing, China). Stained sections were imaged using a Leica DM6B upright microscope.

### Detection of inflammatory cytokines by ELISA

Inflammatory cytokines (TNF-α, IL-1β, IL-6, and IL-10) in colon tissues were quantified using ELISA kits (Meibo Biotechnology, Jiangsu, China) following the manufacturer’s instructions. Optical density was measured at 450 nm using a BioTek microplate reader, and cytokine concentrations were calculated from standard curves.

### Cell viability assay

NCM460 cells were plated in 96-well plates and incubated for 24 h to promote adhesion. The medium was then replaced with RPMI 1640 containing polystyrene (PS) microplastics of different sizes and concentrations. After 24 h of exposure, CCK-8 reagent was added and incubated for 2 h. Absorbance at 450 nm was recorded to assess cell viability.

### Apoptosis detection

Cells were harvested using EDTA-free trypsin, washed with PBS, and centrifuged at 800 rpm for 5 min. Pellets were resuspended in 500 µL binding buffer and transferred to flow tubes. Annexin V-FITC and PI (5 µL each) were added and gently mixed. After 10 min of dark incubation at room temperature, samples were analyzed within 1 h on a Beckman Coulter flow cytometer. FITC and PI signals were collected via FL1 and FL3, and data were processed using FlowJo.

### Live/dead staining assay

NCM460 cells were seeded into 24-well plates and exposed to PS microplastics of various sizes. Calcein AM/PI staining was prepared per instructions. After removing the medium, cells were washed with PBS and incubated with 250 µL stain at 37 °C in the dark for 30 min. Green (Ex/Em = 494/517 nm) and red (Ex/Em = 535/617 nm) fluorescence were observed under a microscope.

### Proteomics sequencing

NCM460 cells were lysed in protease inhibitor buffer and disrupted by sonication on ice. Following centrifugation at 12,000 rpm (4 °C, 15 min), the supernatant was collected and protein concentration was measured using a BCA assay (Biyuntian, Shanghai). Approximately 100 µg of protein was reduced with DTT, alkylated with IAM, and separated via SDS-PAGE. Protein bands of interest were excised and digested with trypsin. Peptides were desalted using C18 columns, eluted with 0.1% formic acid, and dried under nitrogen gas.

Samples were analyzed using an Easy-nLC 1200 system coupled to a Q Exactive HF mass spectrometer (Thermo Scientific). Peptides were reconstituted in 0.1% formic acid and loaded onto a C18 column (150 mm × 75 μm, 3 μm) for gradient elution with 0.1% formic acid and 80% acetonitrile at 300 nL/min. Mass spectra were acquired in positive ion mode across 350–1800 m/z.

Proteomic data were processed using Proteome Discoverer v2.4 with the UniProt database. Differential protein expression was quantified using label-free or TMT-based strategies. Functional enrichment (GO and KEGG) was performed to explore pathways involved in oxidative stress, metabolism, and cell regulation.

### Western blotting

Cells were lysed on ice in buffer containing PMSF and extraction reagent. Protein levels were determined using a BCA kit. Equal amounts were resolved by 10% SDS-PAGE and transferred onto PVDF membranes. After blocking with 5% non-fat milk for 1 h at room temperature, membranes were incubated overnight at 4 °C with primary antibodies targeting Fosl1, P53, Slc7a11, Gpx4, YAP, and GAPDH. Following TBST washes, HRP-labeled secondary antibodies were applied for 1 h at room temperature. Signals were detected via enhanced chemiluminescence using a Tanon imaging system (Shanghai, China).

### Mitochondrial membrane potential detection

JC-1 staining was prepared per instructions. For positive control, cells were treated with 10 µM CCCP for 20 min. After removing the medium, cells in 24-well plates were rinsed with PBS and incubated with 1 mL complete medium plus 1 mL JC-1 solution at 37 °C for 20 min. Meanwhile, 1× JC-1 buffer was prepared on ice.

Staining solution was removed, and cells were washed twice with cold buffer. Fresh medium was added, and fluorescence was observed under an Olympus inverted microscope. Red fluorescence (Ex/Em = 525/590 nm) indicated normal mitochondrial potential; green (Ex/Em = 490/530 nm) reflected depolarization.

### Cell ROS detection

DCF-DA was diluted 1:1000 in serum-free medium to a final concentration of 10 µM, following the manufacturer’s instructions. After removing the original medium, cells were incubated with DCF-DA at 37 °C for 20 min. Excess dye was removed by washing three times with serum-free medium. Rosup was used as a positive control to induce oxidative stress. Fluorescence was observed using an Olympus inverted microscope (Ex/Em = 488/525 nm).

### Tissue Immunofluorescence (IF)

Paraffin-embedded mouse colon tissues were cut into 3 μm sections and deparaffinized with distilled water. Endogenous peroxidase activity was blocked using AR1108 (Boster, Wuhan) for 10 min at room temperature, followed by PBS rinses. Antigen retrieval was carried out via microwave heating. Sections were then incubated in 5% BSA (Biosharp, Hefei) for 2 h at room temperature. Primary antibodies—CD11b (1:200, Huabio), CD8 (1:200, Proteintech), and MHC II (1:200, Huabio)—were applied and incubated overnight at 4 °C. After returning to room temperature, sections were washed with PBS and gently blotted. FITC-labeled secondary antibodies (BL033A, Biosharp) were added and incubated for 2 h in the dark. Following PBS washes, nuclei were counterstained with DAPI in anti-fade mounting medium (P0131, Beyotime). Fluorescence images were acquired using a Leica upright fluorescence microscope.

### CD4/CD8 detection

CD4⁺ and CD8⁺ T cell subsets were assessed by flow cytometry. Spleens were collected and mechanically dissociated to isolate lymphocytes, followed by red blood cell lysis using a commercial buffer. Cells were washed with PBS and transferred into flow tubes. Samples were incubated at 37 °C for 4 h with fluorophore-labeled antibodies: PB450-CD45, FITC-CD4, APC-CD8, and PerCP/Cyanine5.5-CD3. After staining, cells were washed and resuspended in staining buffer. Flow cytometric analysis was conducted using a Beckman Coulter system, and data were analyzed with FlowJo software.

### DC cell detection

Dendritic cell (DC) subsets were analyzed by flow cytometry. Splenic lymphocytes were isolated, washed with PBS, and transferred into flow tubes. Cells were incubated at 37 °C for 4 h with fluorophore-labeled antibodies: PB450-CD45, PE-CD11c, FITC-MHC II, APC-CD80, and PE/Cyanine7-CD86. Following staining, cells were washed and resuspended in staining buffer. Flow analysis was performed on a Beckman Coulter cytometer, and data were processed using FlowJo.

### MDA (Malondialdehyde) content detection

After cell harvesting and centrifugation, 1 mL of extraction buffer was added, and samples were sonicated. Lysates were centrifuged at 8000 g, 4 °C for 10 min, and the supernatant was collected and kept on ice. MDA working solution was prepared per the manufacturer’s protocol. A 200 µL aliquot of supernatant was transferred to a microcuvette or 96-well plate. Absorbance at 532 nm and 600 nm was measured using a spectrophotometer. ΔA values were calculated by subtracting blank readings from sample absorbance. MDA levels were determined using the standard formula.

### Cellular glutathione (GSH) detection

NCM460 cells were seeded in 96-well plates and incubated for 24 h. After medium removal, PS microplastics of different sizes diluted in RPMI-1640 were added and incubated for another 24 h. Cells were rinsed once with PBS to eliminate residual particles. Then, 150 µL of glutathione detection reagent was added per well. Absorbance at 412 nm was measured every 5 min over a 25 min period using a microplate reader. Glutathione concentration was determined based on a standard curve.

### Dual-luciferase reporter assay

Culture cells in good growth condition, and plate them into a 24-well culture plate the day before plasmid transfection. On the day of transfection, carry out plasmid transfection according to the experimental design. After 24 h of transfection, observe the expression of the fluorescently labeled gene in the cells using a fluorescence microscope. Then, treat the cells with the Dual-Luciferase Reporter Assay Kit (RG027, Biyuntian) and perform luciferase expression detection.

### ChIP-qPCR (Chromatin Immunoprecipitation and Quantitative PCR)

First, add adherent cells and suspension cells to culture medium with 1% formaldehyde and PBS solution respectively for cross-linking, incubating at room temperature for 10 min, and then add glycine to terminate the cross-linking. For adherent cells, wash with pre-chilled PBS and scrape the cells; for suspension cells, centrifuge to collect them. After adding lysis buffer, incubate the cells on ice and sonicate them to break up the chromatin. Next, mix the lysate with Protein A/G magnetic beads, and incubate for 3 h. Use a magnetic rack to separate and wash the beads. Then, elute the immunocomplexes from the beads with elution buffer, and perform cross-link reversal in a 65 °C water bath, adding RNase A and Proteinase K for further treatment. Finally, purify the target DNA through DNA extraction and perform qPCR to quantitatively analyze the Ct values and compare differences across groups.

### Molecular Docking

Molecular docking was performed to investigate the potential interaction between Fosl1 (UniProt ID: P15407) and DNA-bound TP53 (Gene ID: 7157). Protein and nucleic acid docking was conducted using AlphaFold3 (https://alphafoldserver.com). Among the predicted complexes, the structure with the highest ranking score was selected for downstream analysis.

Protein preparation was performed in PyMOL v2.4, including removal of water molecules and non-essential ligands, followed by hydrogen atom addition for structure stabilization. The resulting protein–DNA docking models were visualized using PyMOL to analyze spatial conformations and potential binding interactions between Fosl1 and TP53.

### Energy metabolism sequencing

A 50 mg (± 2.5 mg) sample was extracted with 500 µL of pre-chilled 70% methanol (− 20 °C), vortexed for 3 min, and centrifuged at 12,000 rpm, 4 °C for 10 min. After a second centrifugation to remove proteins, 200 µL of the supernatant was filtered and stored at − 20 °C for analysis. Metabolite profiling was performed using UHPLC–MS/MS on a Waters ACQUITY H-Class system with a BEH Amide column (1.7 μm, 100 × 2.1 mm). The mobile phases consisted of 10 mM ammonium acetate with 0.3% ammonia (A) and 90% acetonitrile (B). Separation was achieved with a 0.40 mL/min flow rate at 40 °C and a 2 µL injection volume under the following gradient: 0–1.2 min (5:95), 8 min (30:70), 9–11 min (50:50), 11.1–15 min (5:95). MS analysis was carried out on a QTRAP^®^ 6500 + equipped with an ESI source at 550 °C (+ 5500 V/−4500 V; curtain gas: 35 psi) in MRM mode using optimized acquisition settings. Metabolites were identified via spectral comparison with the MWDB and quantified based on MRM peak areas using external calibration curves.

### Atomic force microscopy (AFM)

Atomic force microscopy (AFM) measurements were performed using a NT-AIST AFM system (HORIBA, Japan) operating in force mapping mode, equipped with high-precision pyramidal probes (MikroMasch, USA). The cantilevers used had a nominal spring constant of 0.5 N/m. After each measurement session, the AFM tip was cleaned with ethanol and sterilized under UV light to remove potential contaminants. Both surface topography and mechanical property (Young’s modulus) maps were acquired to characterize the biological samples. Topographical scans were conducted over a 30 μm × 30 μm area, while force mapping for Young’s modulus was performed over a 10 μm × 10 μm region, during which 400 force–indentation curves were collected and analyzed for modulus calculation.

### Actin-Tracker Red-Rhodamine

Cells were washed with PBS and fixed in 3.7% formaldehyde for 20 min at room temperature. After fixation, they were rinsed three times with 0.1% Triton X-100 in PBS (5 min each). Actin-Tracker Red was diluted 1:40–1:200 in PBS containing 1–5% BSA and 0.1% Triton X-100. A 200 µL volume of staining solution was applied per slide and incubated in the dark for 30–60 min at room temperature. Slides were then washed three times with PBS/Triton X-100 at 5-min intervals. Fluorescence was imaged using a Leica upright microscope.

### Cell Immunofluorescence

NCM460 cells were cultured on sterilized coverslips in 12-well plates and incubated for 12 h to allow attachment. After medium removal, cells were washed with PBS and fixed in 4% paraformaldehyde for 10 min at room temperature, followed by three PBS rinses. Permeabilization was performed using 0.2% Triton X-100 in PBS for 10 min, then washed again. Blocking was done with 5% BSA in PBS for 30 min at room temperature. Cells were incubated with primary antibodies for 1 h in the dark, followed by three 5-min PBS washes. Nuclei were stained with DAPI (1:1000 in PBS) for 1 min in the dark and rinsed. Coverslips were mounted with 90% glycerol, and fluorescence was imaged using a Leica upright microscope.

### Cell lactate content detection

To quantify intracellular lactate, 200 µL of PBS was added to each cell sample and homogenized. Lysates were centrifuged at 10,000 × g, 4 °C for 10 min, and supernatants were collected and kept on ice. Part of the supernatant was used for protein measurement to normalize lactate levels. Lactate assay reagents and standards were prepared following the manufacturer’s protocol. Standards were serially diluted from 0 to 7 mmol/L. For detection, 5 µL of each standard or sample was added to a 96-well plate, followed by 100 µL of enzyme solution and 20 µL of reagent III. After 10 min incubation at 37 °C, 180 µL of reagent IV was added, and the plate was shaken for 5 s. Absorbance at 530 nm was recorded using a microplate reader, and lactate levels were determined from the standard curve.

### ATP content detection

Cells were lysed on ice or at 4 °C using 200 µL of lysis buffer per well (6-well plates). Lysates were pipetted to mix and centrifuged at 12,000 × g, 4 °C for 5 min. Supernatants were collected for ATP analysis. ATP standards ranging from 0.01 to 10 µM were prepared for calibration. The assay reagent was diluted 1:9 to generate the working solution. A total of 100 µL of working solution was added to each well or tube and incubated at room temperature for 5 min. Then, 20 µL of sample or standard was added, gently mixed, and incubated for at least 2 s. Luminescence was detected using a chemiluminescence reader, and ATP concentrations were calculated from RLU values using the standard curve.

### Extracellular acidification rate (ECAR) detection

To evaluate ECAR, cells were trypsinized, neutralized with serum-containing medium, washed with saline, and pelleted by centrifugation. After removing the supernatant, cells were resuspended in Reagent 1 as instructed. A 100 µL cell suspension was dispensed into each well of a black 96-well plate; blank wells received 100 µL of Reagent 1. Plates were incubated at 37 °C in the dark for 30 min. Subsequently, 100 µL of ECAR working solution was added. Fluorescence (Ex/Em = 490/535 nm) was recorded every 2–5 min for 100–120 min using a microplate reader. ECAR was determined by calculating the fluorescence change rate (ΔF/ΔT) over time.

### Statistical analysis

Data are presented as mean ± SEM. Statistical analysis was performed using GraphPad Prism 10.1. One-way ANOVA followed by Tukey’s post hoc test was used for group comparisons. *p* < 0.05 was considered statistically significant.

## Results

### Characterization of PS microplastics and uptake of microplastics of different sizes by intestinal epithelial cells

**Fig. 1:**
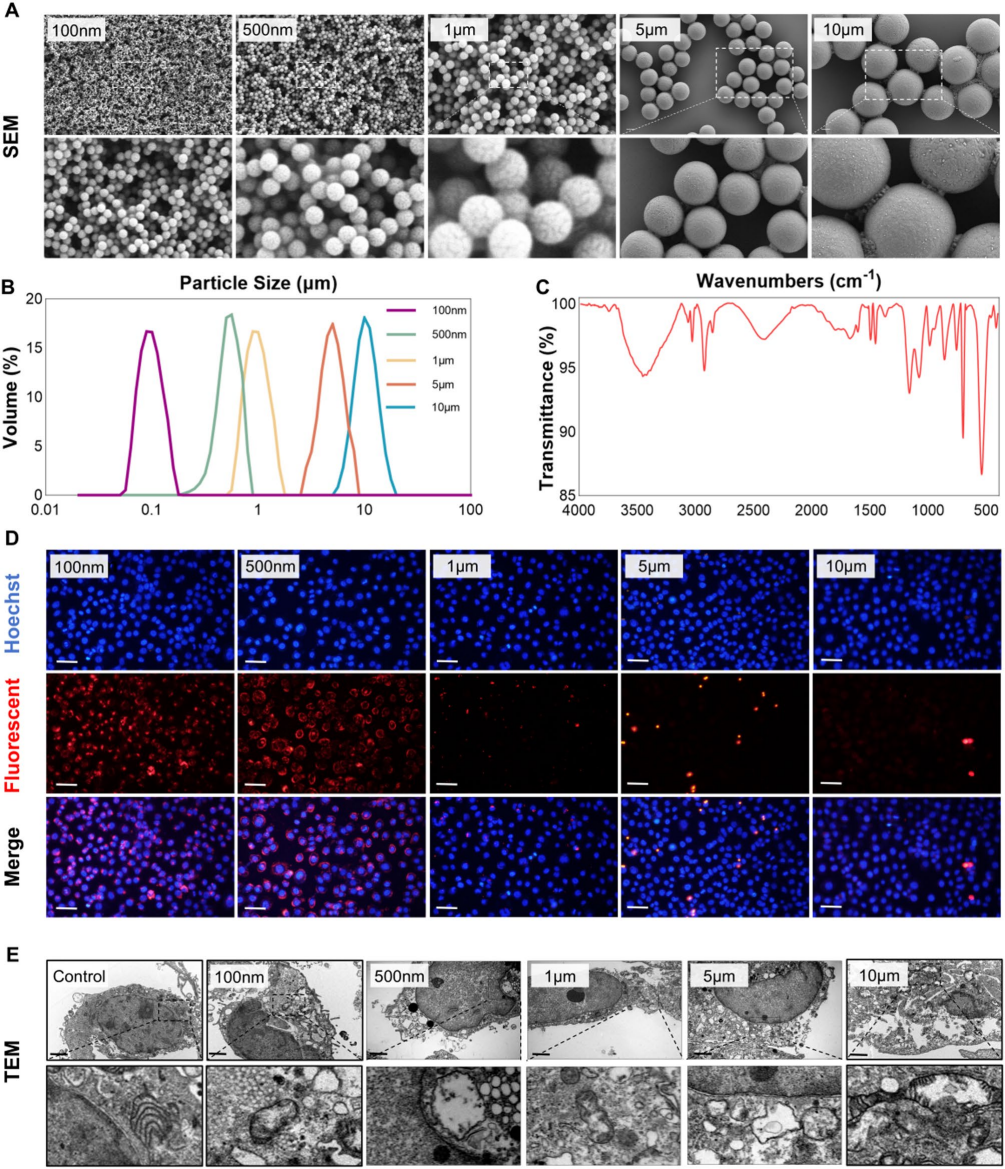
Characterization of PS microplastics and uptake of microplastics of different sizes by intestinal epithelial cells. **A** SEM images of five different diameters of PS microplastics at low and high magnifications. Scale bar = 2 μm. (**B**) Laser particle size analysis of the average sizes and distribution of the five different sizes of PS microplastics. (**C**) FTIR spectrum analysis of PS microplastics. (**D**) Co-culturing five different sizes of red fluorescent PS microplastics with NCM460 cells for 24 h, observing the uptake of PS microplastics by cells. Images show DAPI channel, red fluorescence channel, and merged images from top to bottom. Scale bar = 100 μm. (**E**) Co-culturing five different sizes of PS microplastics with NCM460 cells for 24 h, observing the uptake of PS microplastics by cells using TEM images at low and high magnifications. Scale bar = 2 μm

As characterized by SEM and FTIR, the PS microplastics used in this study were spherical and chemically consistent (Fig. 1A–C).

To examine whether microplastics of different sizes could be internalized by human intestinal epithelial cells (NCM460), we cultured NCM460 cells in vitro. The results indicated that microplastics sized 100 nm and 500 nm were readily engulfed by NCM460 cells and accumulated in large quantities within the cells. In contrast, 1 μm and 5 μm microplastics were minimally internalized, and 10 μm microplastics were not taken up by the cells at all (Fig. 1D). To further confirm these observations, we employed transmission electron microscopy (TEM) to observe the uptake of microplastics by the cells. TEM imaging revealed that NCM460 cells ingested a significant number of 100 nm and 500 nm microplastic particles, while only a few 1 μm and 5 μm microplastic particles were internalized, and no internalization of 10 μm particles was observed (Fig. 1E).

Additionally, we examined the effects of microplastic uptake on cellular integrity. In the 100 nm, 500 nm, and 1 μm experimental groups, the continuity and integrity of the cell membranes remained largely intact, although some damage to the mitochondria was noted. In contrast, in the 5 μm and 10 μm experimental groups, the integrity of the cell membranes was compromised, while mitochondrial morphology remained relatively normal, suggesting that mechanical damage to the cells occurred to a certain extent.

### Long-term exposure to PS microplastics induces Colon inflammation in mice

**Fig. 2:**
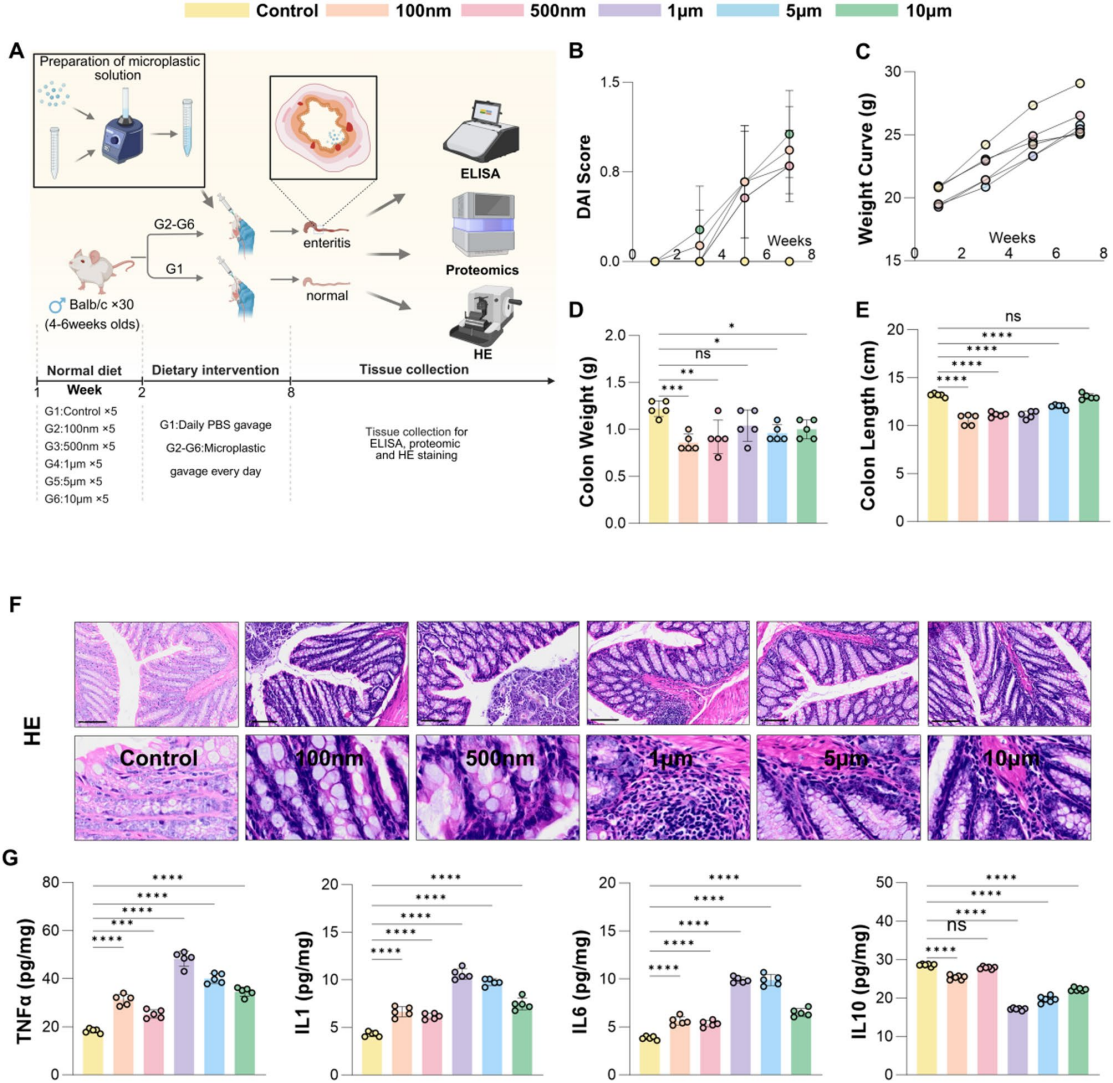
Long-term exposure to PS microplastics induces colon inflammation in mice. **A** Schematic diagram of the treatment process for microplastic and control groups. (**B**) DAI scores during the gavage period for the microplastic and control groups. (**C**) Changes in body weight gain curves for the microplastic and control groups during the gavage period. (**D**) Comparison of colon weight between control and microplastic-treated groups. (**E**) Comparison of colon length between control and microplastic-treated groups. (**F**) Anatomical diagram of the intestines and H&E staining of colon tissue in the microplastic and control groups. (**G**) Expression levels Four inflammatory factors in the microplastic and control groups (ELISA). *p < 0.05, **p < 0.01, ***p < 0.001, ****p < 0.0001

After a one-week acclimation, BALB/c mice were randomly divided into six groups: one control and five polystyrene microplastic (PS-MP) exposure groups. The treated groups received daily oral gavage of 1 mg/mL PS-MP suspension, while controls received equal volumes of PBS. After seven weeks of treatment and care, mice were sacrificed, and colon tissues, serum, and fecal samples were collected for ELISA, H&E staining, and other biochemical assays (Fig. 2A).

To evaluate colon inflammation, mice were dynamically monitored using a simplified Disease Activity Index (DAI) scoring system (Fig. 2B) while body weight changes were recorded (Fig. 2C, Additional file 1: Fig. S1). At baseline, no significant differences in body weight were observed between the microplastic-exposed and control groups (*P* > 0.05). However, during the gavage period, mice exposed to microplastics exhibited a slower rate of weight gain compared to controls.By the end of the study, mice exposed to microplastics showed a significant reduction in body weight compared to controls (*P* < 0.05).

Further analysis of colon weight and length revealed a significant reduction in the microplastic-exposed groups compared to controls, with the most pronounced effect observed in the 100 nm group (*P* < 0.05, Fig. 2D and E, Additional file 2: Fig. S2). Additionally, H&E staining of colon tissue sections indicated marked inflammatory cell infiltration across all microplastic-treated groups (Fig. 2F). ELISA analysis further demonstrated a significant increase in pro-inflammatory cytokines (TNF-α, IL-1β, and IL-6) alongside a notable reduction in the anti-inflammatory cytokine IL-10 (*P* < 0.05, Fig. 2G).

These results indicate that long-term exposure to PS microplastics impairs body weight gain and induces colon inflammation in mice.

### Microplastics induce Colon epithelial cell death

**Fig. 3:**
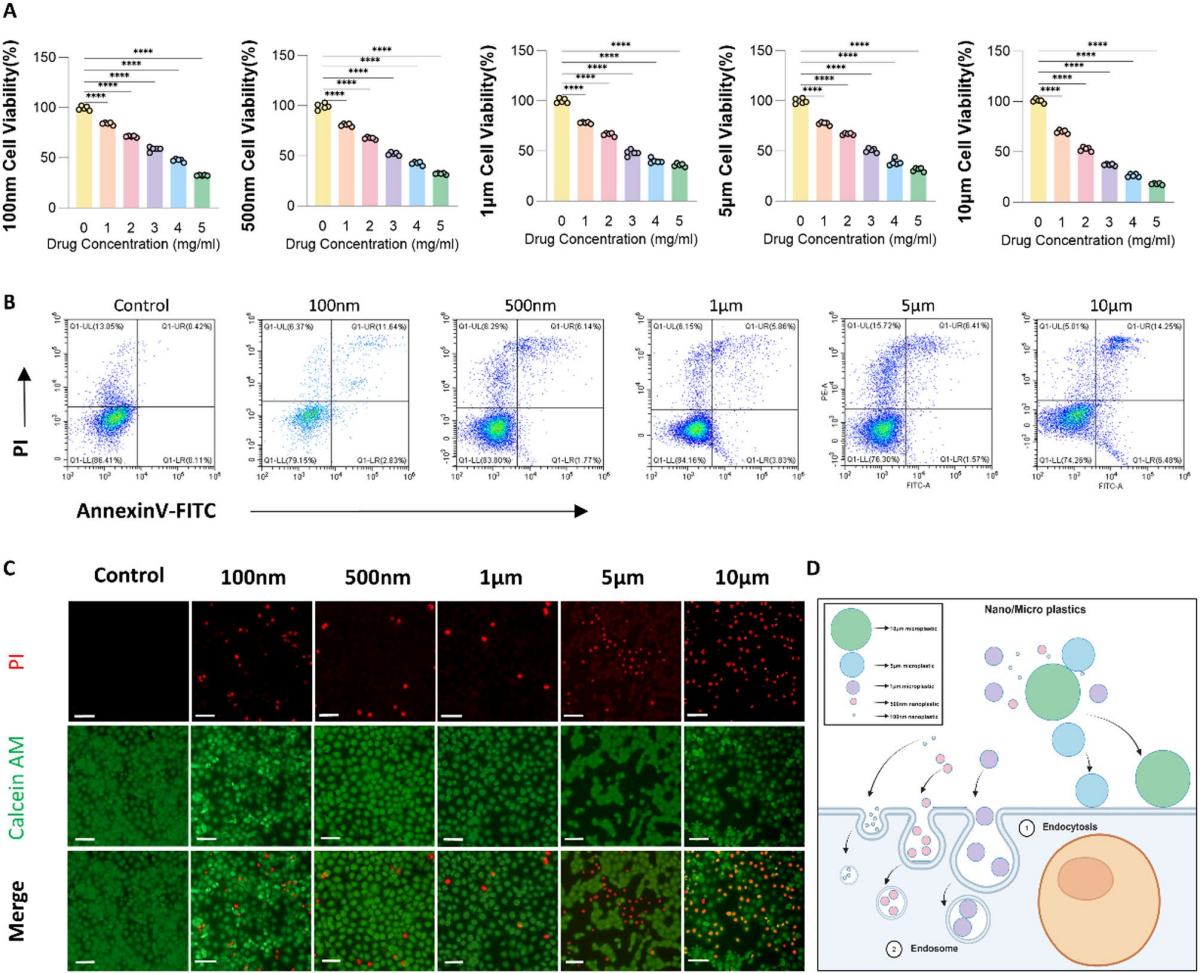
Microplastics induce colonic epithelial cell death. (**A**) CCK-8 cell proliferation and toxicity assay. (**B**) Annexin V-FITC flow cytometry apoptosis detection. (**C**) Calcein-AM/PI live-dead cell fluorescence staining. Scale bar = 100 μm. (**D**) Model diagram of NCM460 cells ingesting PS microplastics of different sizes. *p < 0.05, **p < 0.01, ***p < 0.001, ****p < 0.0001

The previous results suggested that long-term exposure to PS microplastics induces colon inflammation in mice. To further investigate the mechanisms underlying cellular damage caused by microplastics, we selected the NCM460 cell line for in vitro analysis. First, to determine the appropriate concentration of PS microplastics for the experiment, we performed CCK-8 cell proliferation and toxicity assays. As shown in Fig. 3A, treatment with PS microplastics of varying sizes exhibited concentration-dependent cytotoxicity. At a concentration of 1 mg/mL, PS microplastics caused mild damage to NCM460 cells, with similar cell viability across all particle size groups. Specifically, cell viability was 84%, 80.8%, 78.2%, 77.4%, and 70% for the 100 nm, 500 nm, 1 μm, 5 μm, and 10 μm groups, respectively. However, at higher concentrations, all groups exhibited significant cell damage, with a noticeable decrease in cell viability.

To avoid excessive cell death caused by high concentrations of PS microplastics and to better explore the size-dependent effects of microplastics on cellular damage, we selected 1 mg/mL as the baseline concentration for subsequent experiments. Thus, in the following cell experiments, the Control group served as the control, and the PS microplastic concentration in all size groups was 1 mg/mL.

Figure 3B presents the apoptosis analysis, indicating an apoptosis rate of 0.42% in the control group, compared to 8.42% (100 nm group), 6.14% (500 nm group), 5.86% (1 μm group), 6.41% (5 μm group), and 14.25% (10 μm group). Figure 3C illustrates Calcein-AM/PI live-dead cell fluorescence staining, where live cells exhibited green fluorescence (Calcein-AM), while dead cells displayed red fluorescence (PI). The red-to-green fluorescence ratio served as an indicator of cell damage. In the control group, minimal red fluorescence was observed, indicating normal cell viability. In contrast, microplastic exposed groups exhibited increased red fluorescence, suggesting varying degrees of cytotoxicity. Notably, the 100 nm and 10 μm groups showed a more pronounced increase in red fluorescence compared to the 500 nm, 1 μm, and 5 μm groups, indicating that cell damage was most severe in the 100 nm and 10 μm groups. Furthermore, the substantial size difference between 100 nm and 10 μm polystyrene microplastics may contribute to their distinct biological effects.

Based on these results, we observed that PS microplastics smaller than 1 μm were more readily taken up by NCM460 cells, whereas PS microplastics larger than 1 μm were less efficiently internalized. The most notable effects were observed with the 100 nm and 10 μm PS microplastics. Therefore, we selected 100 nm and 10 μm PS microplastics as representative particle sizes for further investigation into their potential mechanisms of cellular damage (Fig. 3D).

### Proteomics reveals mechanistic differences in Colon inflammation induced by different sizes of PS microplastics

**Fig. 4:**
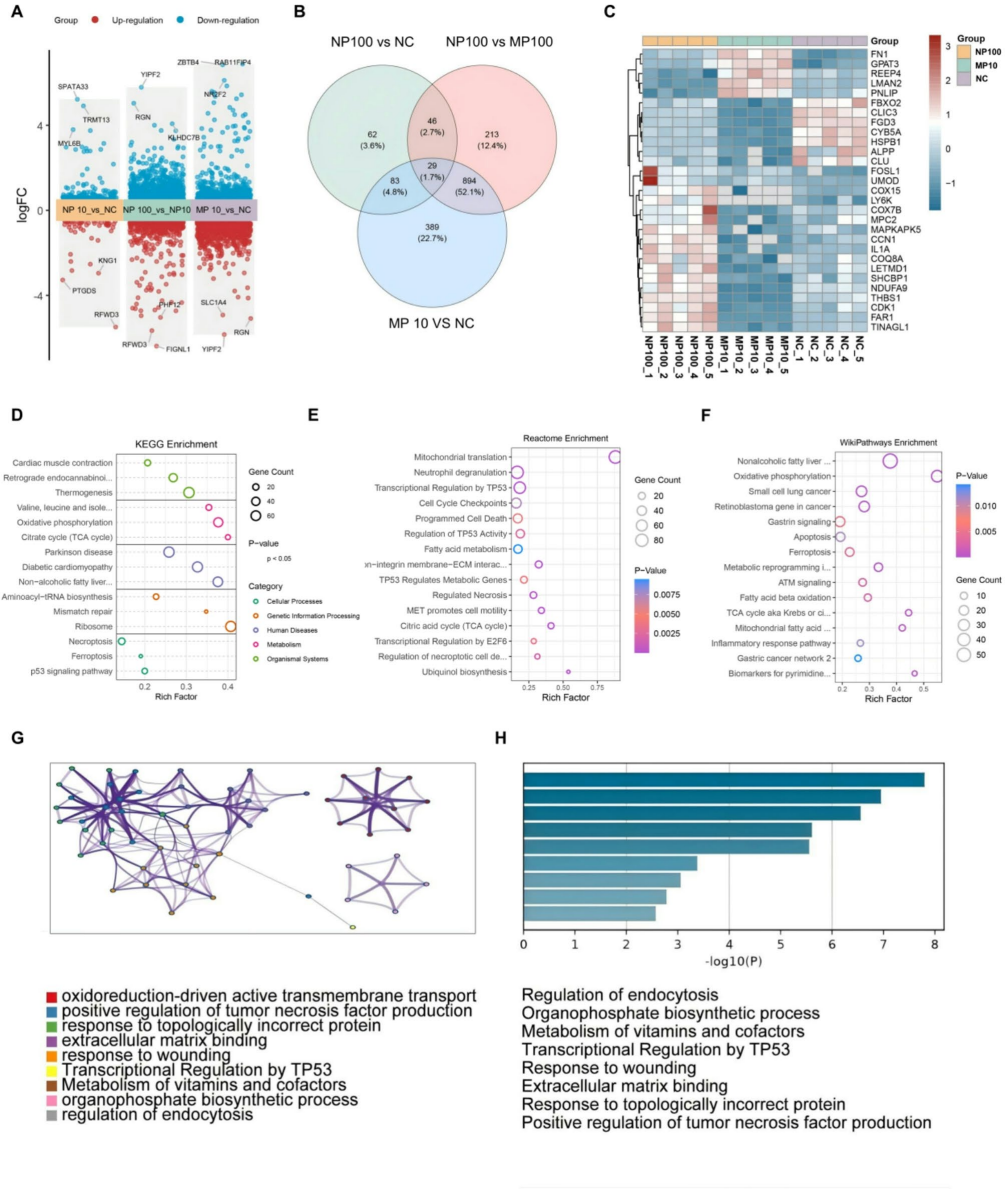
Proteomics reveals mechanistic differences in colon inflammation induced by different sizes of PS microplastics. (**A**) Volcano plots illustrating differential gene expression in NP10 vs. NC, NP100 vs. NP10, and MP10 vs. NC comparisons. Red and blue dots indicate upregulated and downregulated genes, respectively. Significantly changed genes are annotated. (**B**) Venn diagram showed the overlap of three groups of differential genes: NP_10 vs. NC, NP_100 vs. NP_10, MP_10 vs. NC. (**C**)Heatmap displaying expression profiles of DEGs in NP100, MP10, and NC groups. Color gradients reflect expression levels (red: high, blue: low). Hierarchical clustering revealed group-specific gene expression patterns. (**D**) KEGG pathway enrichment analysis. The KEGG enrichment bubble map showed the signaling pathway of significant enrichment of differential genes. (**E**) Reactome pathway enrichment analysis. Reactome bubble map showed the enrichment of differential genes in the Reactome pathway. (**F**) WikiPathways enrichment analysis. Bubble plot illustrates significant enrichment of DEGs across biological pathways. The X-axis shows the Rich Factor (DEG ratio per pathway), Y-axis displays pathway names. Bubble size denotes gene count; color indicates p-value.(**G**) Gene function network analysis, where the network maps showed significant associations between enrichment functions and pathways. Nodes represent enrichment functions, and lines indicate the degree of similarity or gene sharing between functions or pathways. (**H**) Summary bar chart of significant function, which summarizes the functional and biological processes of significant enrichment

To further explore the mechanisms by which PS microplastics of different sizes damage intestinal epithelial cells, we selected 100 nm and 10 μm particles as representative sizes. These particles were co-cultured with NCM460 cells for 24 h, after which proteomic sequencing analysis was conducted (Fig. 4A-H). The results revealed significant differences in gene expression between the NP group (100 nm PS microplastics) and the MP group (10 μm PS microplastics) (Fig. 4A). Venn diagram analysis (Fig. 4B) showed that 29 differentially expressed genes were common to both groups, while the NP100 and MP10 groups had 62 and 389 unique differentially expressed genes, respectively. This suggests that PS microplastics of different sizes may induce cellular damage through distinct molecular mechanisms.

Heatmap analysis (Fig. 4C) identified the Fosl1 gene, which is associated with ferroptosis, as significantly upregulated in the NP100 group. Other genes related to cellular stress and metabolism, such as HSPB1 and MAPKAPK5, were also elevated, indicating that smaller-sized microplastics may induce oxidative stress and inflammatory responses. In contrast, the MP 10 group exhibited significant changes in metabolism-related genes, such as a marked increase in the expression of GPAT3 and FGD3. Additionally, MP 10 group showed alterations in genes related to mitochondrial function and energy metabolism, including COX15 and MPC2, suggesting a link to energy metabolism remodeling and cell structural damage in intestinal epithelial cells.

KEGG pathway enrichment analysis further elucidated the potential mechanisms of action for the two sizes of PS microplastics (Fig. 4D). Differential genes in the NP group were predominantly enriched in ferroptosis and P53 signaling pathways, indicating that smaller-sized PS microplastics may induce ferroptosis through lipid peroxidation. In contrast, differential genes in the MP group were notably enriched in metabolic pathways, such as the TCA cycle and oxidative phosphorylation, suggesting that larger-sized PS microplastics may induce cell death by modulating glycolysis and metabolic remodeling.

Reactome pathway analysis (Fig. 4E) supported these findings, showing that genes in the NP100 group were significantly enriched in programmed cell death and P53 regulation pathways, while those in the MP10 group were concentrated in fatty acid metabolism and mitochondrial function pathways. WikiPathways analysis (Fig. 4F) further highlighted the association of the NP100 group with ferroptosis, while the MP10 group’s genes were enriched in inflammatory response and glycolysis pathways. Gene functional enrichment network analysis (Fig. 4G) indicated that smaller-sized PS microplastics are involved in ferroptosis and P53 signaling, whereas larger-sized PS microplastics mediate cell damage through fatty acid metabolism and glycolysis abnormalities following mechanical damage.

Finally, the functional enrichment bar chart (Fig. 4H) quantified the enrichment levels of significant pathways, underscoring the close association of the NP100 group with ferroptosis and P53 signaling regulation, as well as the significant enrichment of the MP10 group in glycolysis, fatty acid metabolism, and extracellular matrix binding. These results suggest that smaller-sized PS microplastics may cause cellular damage through lipid peroxidation and ferroptosis, while larger-sized PS microplastics likely affect cell function through mechanical damage and metabolic remodeling.

### Small-sized NP-mediated in vitro ferroptosis

**Fig. 5:**
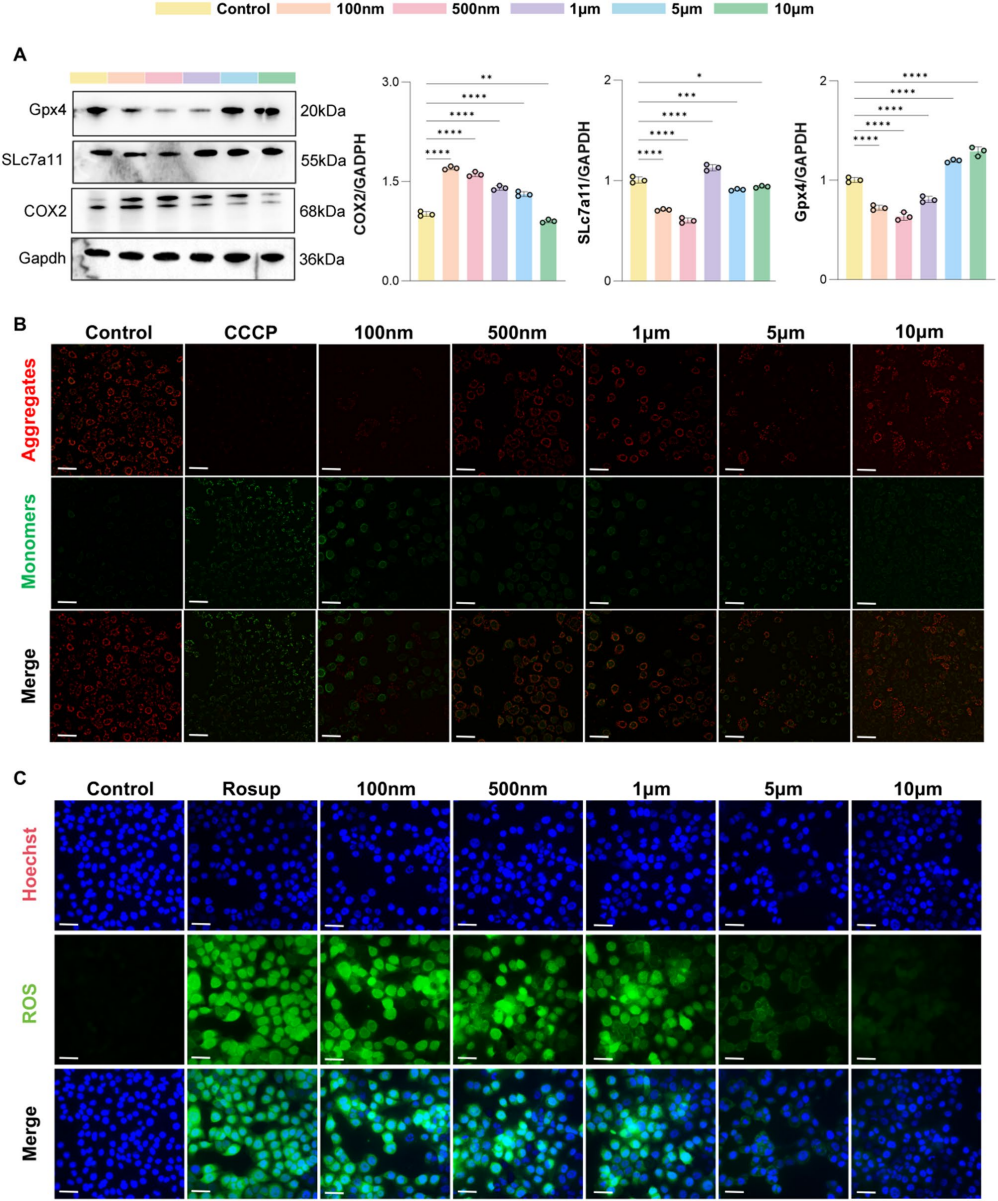
NPs mediated ferroptosis in vitro. (**A**) Western blotting analysis of ferroptosis-related proteins. (**B**) Reactive oxygen species (ROS) assay. Scale bar = 200 μm. (**C**) JC-1 mitochondrial membrane potential assay. Scale bar = 200 μm.*p < 0.05, **p < 0.01, ***p < 0.001, ****p < 0.0001

The proteomic analysis suggested that small-sized PS nanoplastics may primarily induce ferroptosis through lipid peroxidation, while larger-sized PS microplastics are likely to induce cell apoptosis due to metabolic alterations. To validate that nanoplastics can induce ferroptosis in vitro, we assessed the expression of ferroptosis-related protein markers under treatment with different sizes of microplastics (Control, 100 nm, 500 nm, 1 μm, 5 μm, 10 μm). Western blot analysis showed that 100 nm and 500 nm PS microplastics significantly downregulated the expression of ferroptosis-related proteins SLc7a11 and Gpx4, while the 1 μm group exhibited a non-significant downward trend. In contrast, no significant changes were observed in the larger-sized microplastic groups (5 μm and 10 μm) (Fig. 5A).

JC-1 staining was conducted to evaluate mitochondrial membrane potential (MMP) alterations, serving as an indicator of oxidative stress and ferroptosis-associated mitochondrial dysfunction. As shown in Fig. 5B, the Control group predominantly exhibited red fluorescence, with minimal green fluorescence, indicative of intact mitochondrial function. In contrast, the CCCP-positive control group, known to induce mitochondrial dysfunction, displayed a predominance of green fluorescence with minimal red fluorescence, confirming MMP loss. In the 100 nm, 500 nm, and 1 μm groups, a decrease in red fluorescence and a corresponding increase in green fluorescence were observed compared to the Control group, suggesting mitochondrial membrane potential disruption. This effect was less pronounced in the 5 μm and 10 μm groups, further indicating that smaller-sized PS microplastics induce greater mitochondrial dysfunction.

Additionally, we assessed reactive oxygen species (ROS) levels using a ROS assay, where the intensity of green fluorescence corresponds to ROS levels within the cells. The results in Fig. 5C showed that the Control group exhibited minimal green fluorescence, while the ROS inducer (Rosup) positive control group displayed abundant green fluorescence. Among the microplastic-treated groups, the 100 nm, 500 nm, and 1 μm groups exhibited strong green fluorescence, indicating significantly elevated ROS levels. Conversely, the 5 μm and 10 μm groups showed only a slight increase in fluorescence compared to the Control group, suggesting that ROS levels were substantially elevated only in the small-sized PS microplastic-treated groups.

### Ferrostatin-1 alleviates colon inflammation induced by NPs in vivo

In vitro experiments suggested that NPs can induce ferroptosis, contributing to colon inflammation. However, it remained unclear whether this mechanism occurs in vivo and whether ferroptosis inhibition could mitigate NP-induced intestinal damage. Ferrostatin-1 (Fer-1), a well-established ferroptosis inhibitor, is known to suppress lipid peroxidation and oxidative stress. To test its protective effects in vivo, Fer-1 was administered to mice following NP exposure.

**Fig. 6:**
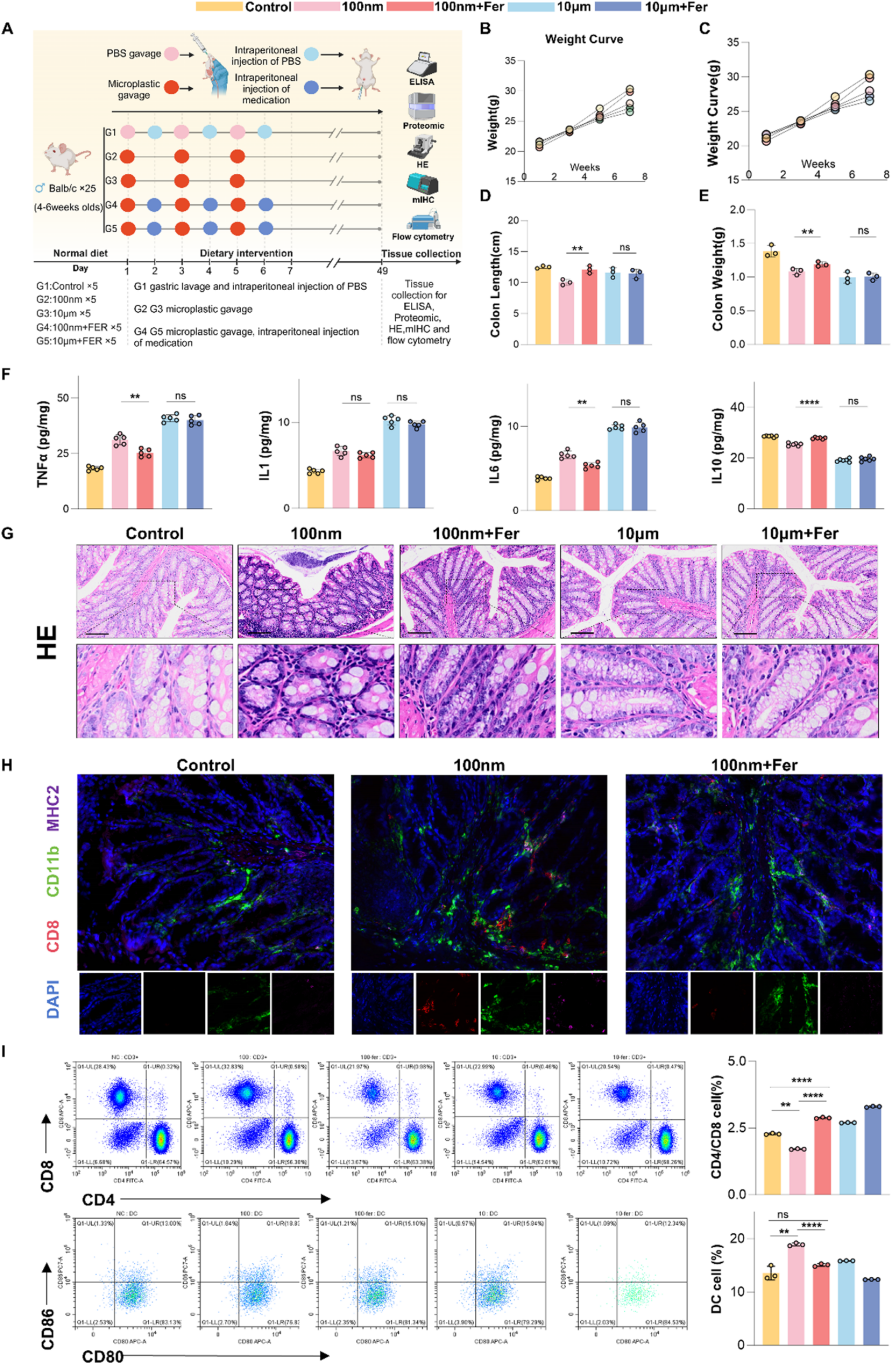
Ferrostatin-1 alleviates colon inflammation induced by NPs in vivo. (**A**) Diagram of the treatment process for microplastic and control groups of mice. (**B**) DAI scores during the gavage process in the microplastic and control groups. (**C**) Body weight growth curves of microplastic and control group mice during gavage. (**D**) Comparison of colon weight in microplastic and control groups. (**E**) Comparison of colon length in microplastic and control groups. (**F**) Expression levels of Four inflammatory factors in microplastic and control groups (ELISA). (**G**) Anatomical diagram and H&E staining of colonic tissue in microplastic and control groups. (**H**) Multiplex immunofluorescence. (**I**) Flow cytometry analysis of CD4/CD8 ratio and DC cells. *p < 0.05, **p < 0.01, ***p < 0.001, ****p < 0.0001

After one week of acclimatization, BALB/c mice were randomly assigned to five groups: a control group, two NP exposure groups (100 nm and 10 μm), and two NP exposure groups receiving intraperitoneal Fer-1. Mice received daily oral gavage of 1 mg/mL PS microplastics, with or without Fer-1, for seven weeks. Colon tissues, serum, and fecal samples were collected for ELISA, H&E staining, and multiplex immunofluorescence (Fig. 6A).

NP exposure significantly reduced body weight, colon length and weight, and increased DAI scores and pro-inflammatory markers, indicating intestinal inflammation. In contrast, Fer-1 treatment effectively improved these parameters (Figs. 6B–F, Additional file3: Fig.S3,Additional file4: Fig.S4 ), suggesting mitigation of NP-induced injury.

Histopathological analysis (Fig. 6G) revealed extensive inflammatory infiltration in NP-treated mice, which was notably alleviated by Fer-1. However, Fer-1 had little effect on large-sized MP-induced inflammation, indicating that ferroptosis may not be involved in the damage caused by larger particles.

To investigate ferroptosis-mediated immune activation, we analyzed colon tissues via multiplex immunofluorescence and flow cytometry. NP-treated mice showed increased infiltration of dendritic cells (DCs) and CD8⁺ T cells, alongside a reduced CD4/CD8 ratio and elevated DC (CD80⁺CD86⁺) counts—hallmarks of excessive immune activation. Fer-1 reversed these changes (Figs. 6H–I), indicating that ferroptosis contributes to immunogenic cell death (ICD) in the colon.

Together, these findings demonstrate that small-sized NPs induce intestinal inflammation through ferroptosis-mediated ICD, and that inhibition of ferroptosis by Fer-1 can effectively alleviate this damage.

### Fosl1 regulates P53 transcription to promote nano-plastics-Induced ferroptosis

Proteomic analysis revealed significant gene expression changes in the NP-treated group, particularly upregulation of Fosl1, Cox-15, and CCN1. Functional enrichment analysis showed that these differentially expressed proteins were enriched in the ferroptosis and p53 signaling pathways. Among them, Fosl1—a member of the FOS transcription factor family and a core component of the AP-1 complex—was identified as a key regulatory molecule potentially linking NP exposure to ferroptosis. Given its involvement in cell proliferation, apoptosis, and stress responses, Fosl1 was selected for further investigation.

**Fig. 7:**
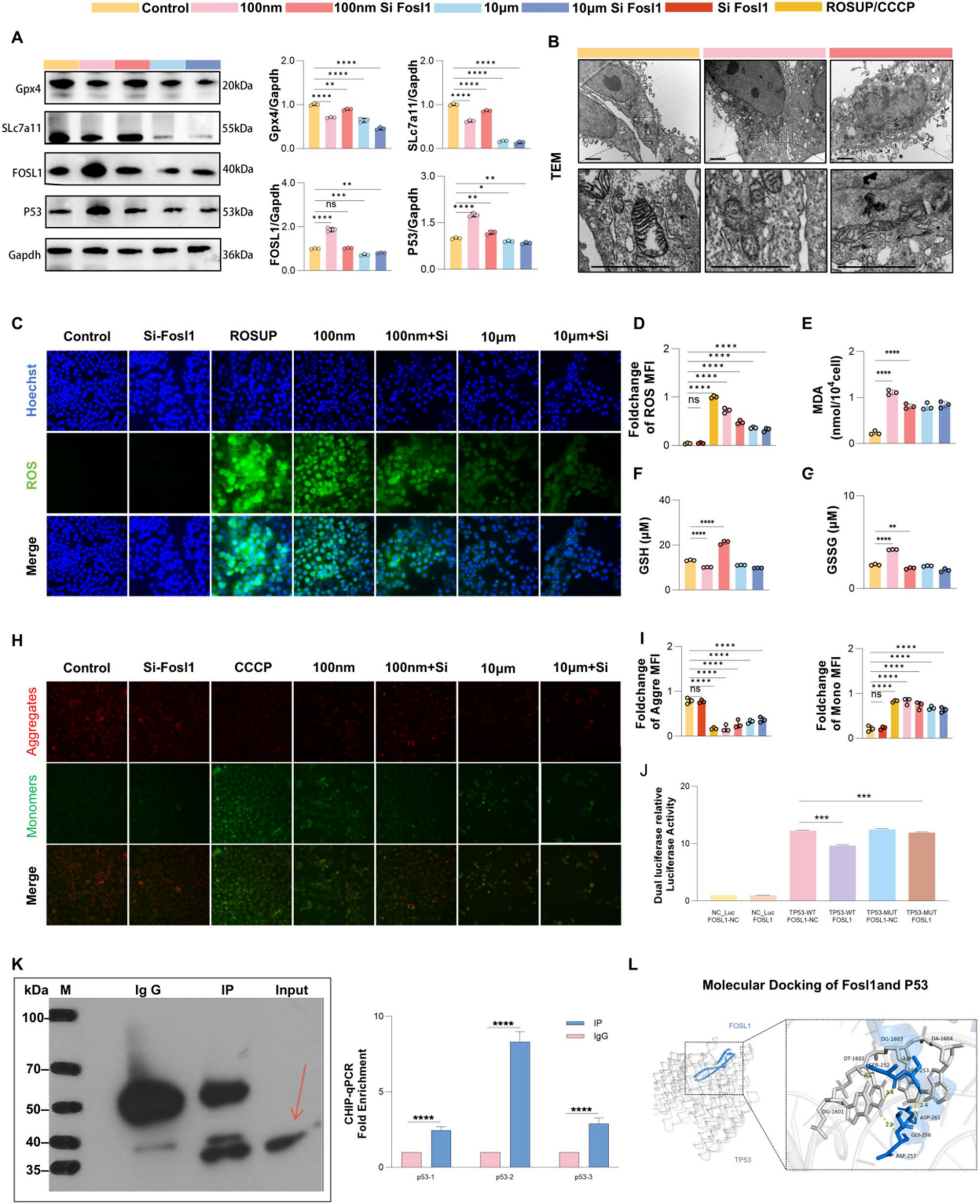
Fosl1 regulates P53 transcription to promote nano-plastics-induced ferroptosis. (**A**) Western blot detection of ferroptosis-related proteins. (**B**) TEM images of mitochondrial changes in NCM460 cells in the Control, 100 nm, and 100 nm + siFOSL1 groups at low and high magnifications. Scale bar = 2 μm. (**C**) ROS detection. Scale bar = 200 μm. (**D**) Fluorescent quantification of ROS detection. (**E**) MDA content detection. (**F**) GSH content detection. (**G**) GSSG content detection. (**H**) JC-1 mitochondrial membrane potential detection. Scale bar = 200 μm. (**I**) Quantification of JC-1 fluorescence in mitochondrial membrane potential detection. (**J**) Dual-luciferase assay. (**K**) Chromatin immunoprecipitation (ChIP)-qPCR experiment. (**L**) Molecular docking analysis. Hydrogen bonds are represented in yellow. FOSL1 and DNA-TP53 are represented in blue and gray, respectively. *p < 0.05, **p < 0.01, ***p < 0.001, ****p < 0.0001

To assess its role in ferroptosis, we conducted in vitro experiments with five treatment groups: Control, 100 nm, 100 nm + siFosl1, 10 μm, and 10 μm + siFosl1. Western blotting (Fig. 7A, Additional file5:Fig.S5) confirmed upregulation of Fosl1 in the 100 nm NP group, along with downregulation of Slc7a11 and Gpx4 upon Fosl1 knockdown—an effect not observed in the 10 μm group.

TEM (Fig. 7B) revealed typical mitochondrial damage in the NP group, including condensed membranes, shrunken volume, disrupted outer membranes, and loss of cristae. Knockdown of Fosl1 mitigated these structural abnormalities, suggesting a protective effect on mitochondrial integrity.

We further evaluated ferroptosis-related biomarkers: ROS (Fig. 7C), mitochondrial membrane potential (JC-1, Fig. 7H), malondialdehyde (MDA, Fig. 7E), and glutathione (GSH/GSSG, Fig. 7F and G). Compared to the 100 nm NP group, the 100 nm + siFosl1 group showed significantly reduced ROS, restored mitochondrial potential, decreased MDA levels, and a shift in GSH/GSSG ratio—indicating reduced oxidative stress and ferroptosis. These changes were not seen in the 10 μm MP groups, suggesting a size-specific effect.

To determine whether Fosl1 directly regulates P53 transcription, we conducted dual-luciferase reporter assays (Fig. 7J) and ChIP-qPCR (Fig. 7K). Results confirmed direct binding of Fosl1 to the P53 promoter. Additionally, molecular docking using AlphaFold3 (Fig. 7L) supported this interaction, providing a structural basis for Fosl1-mediated transcriptional regulation.

Collectively, these findings demonstrate that Fosl1 promotes ferroptosis in NP-induced colon inflammation by enhancing P53 transcription and suppressing Slc7a11 and Gpx4. In contrast, these effects were absent in the MP group, further highlighting the size-dependent mechanism of toxicity.

### Non-targeted energy metabolomics reveals MPs induce colon inflammation via metabolic reprogramming

Proteomic analyses revealed that differentially expressed proteins in the 10 μm microplastic-treated group were predominantly enriched in metabolic pathways, particularly the tricarboxylic acid (TCA) cycle and oxidative phosphorylation. To further explore the impact of large polystyrene microplastics on intestinal epithelial cell metabolism, an energy metabolomics analysis was conducted on colon tissues from both the control and 10 μm microplastic-treated groups. The analysis focused on key metabolic pathways, including glycolysis, the TCA cycle, and oxidative phosphorylation, to elucidate potential metabolic alterations.

**Fig. 8:**
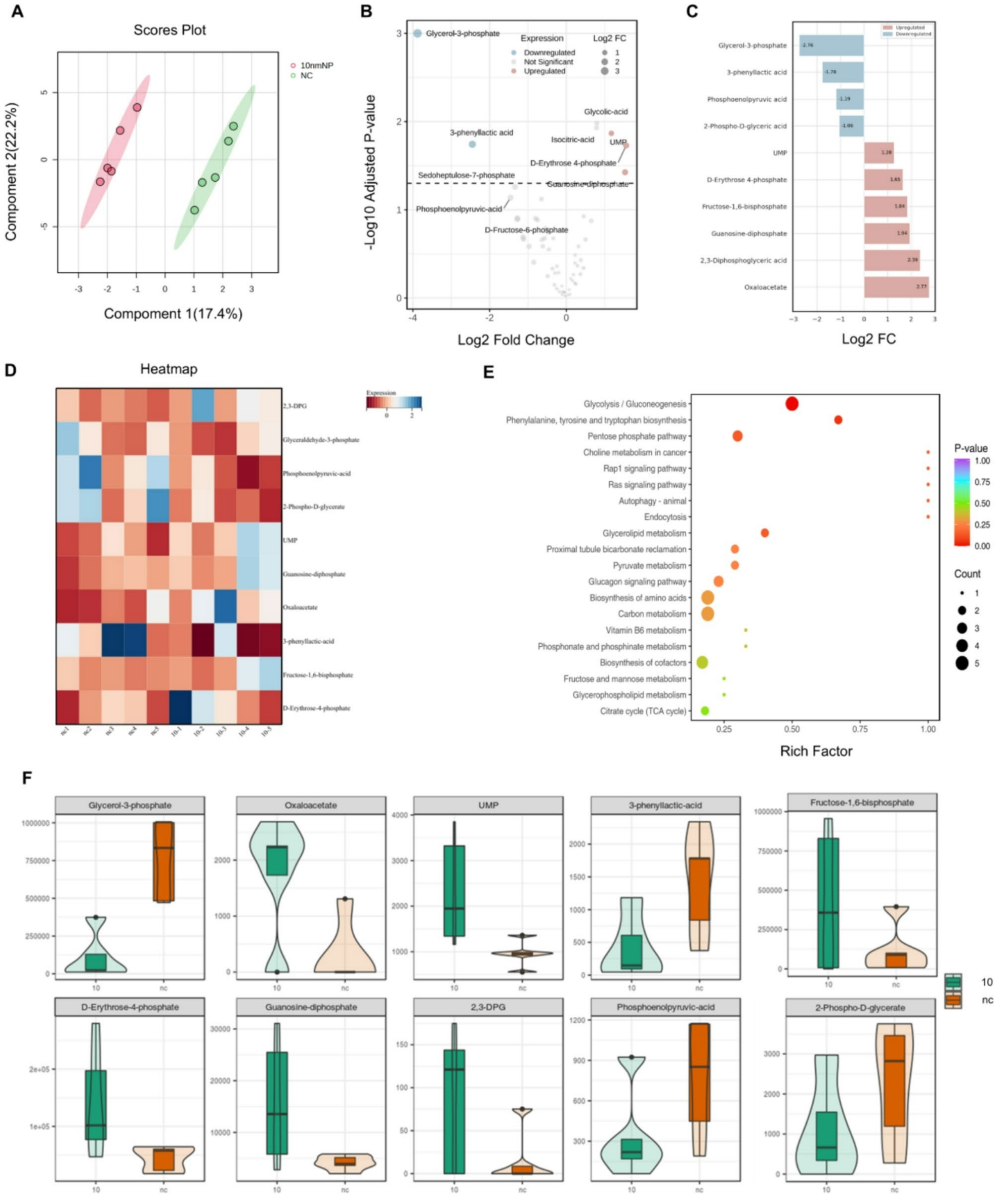
Non-targeted energy metabolomics reveals MPs induce colon inflammation via metabolic reprogramming. (**A)** Principal component analysis (PCA) of sample groups. Component 1 and Component 2 represent the first and second principal components, respectively, with percentages indicating the variance explained. Each point denotes a sample, and colors represent sample groupings. (**B**) Each dot represents a metabolite. Red and blue indicate upregulated and downregulated metabolites, respectively; gray denotes non-significant changes. (**C**) Bar plot of differential metabolites. The X-axis shows logFC values; the Y-axis lists metabolites. Red and blue bars indicate up- and downregulated metabolites, respectively. (**D**) Heatmap of metabolite expression. The horizontal axis shows sample names, and the vertical axis lists metabolites. Expression values are standardized, with red indicating higher and blue indicating lower expression levels. (**E**) KEGG pathway enrichment analysis based on differential metabolites. The Rich Factor indicates the ratio of differential to total annotated metabolites within each pathway; higher values reflect greater enrichment. (**F**) Violin plot illustrating data distribution. The box shows the interquartile range, the thin line represents the 95% confidence interval, the horizontal line indicates the median, and the outer contour reflects density

Energy metabolomics analysis, as demonstrated by principal component analysis (PCA) (Fig. 8A), revealed a clear separation between the MP-treated group and the control group, indicating that MP exposure significantly altered the cellular metabolic profile. Further analysis using volcano plots of differential metabolites (Fig. 8B) identified notable metabolic shifts induced by MP exposure. Specifically, early-stage glycolytic metabolites, such as fructose-1,6-bisphosphate, were significantly upregulated, whereas late-stage glycolytic intermediates, including phosphoenolpyruvate and 2-phospho-D-glycerate, were markedly downregulated. These findings suggest an increase in glycolytic flux accompanied by a metabolic blockade in late-stage glycolysis.

Moreover, a significant upregulation of oxaloacetate, a key intermediate in the TCA cycle, indicates that cells may attempt to compensate for the glycolytic blockade by enhancing TCA cycle activity. However, the diminished late-stage glycolysis alongside the increased TCA cycle activity suggests a potential shift toward anaerobic metabolism.

To further illustrate the changes in metabolite abundance, we presented the variations in key metabolites between the treatment and control groups using bar charts (Fig. 8C), heatmaps (Fig. 8D), and violin plots (Fig. 8F). These comparative analyses revealed that in the large-particle PS microplastic treatment group, glycerol-3-phosphate and phosphoenolpyruvate were significantly reduced, while fructose-1,6-bisphosphate and metabolites from the pentose phosphate pathway, such as D-erythrose 4-phosphate, were significantly elevated. These changes suggest that late-stage glycolytic metabolites were significantly downregulated, and cells may adapt to metabolic stress by enhancing early glycolysis and the pentose phosphate pathway.

Overall, the trend suggests a metabolic shift from aerobic to anaerobic pathways, characterized by increased anaerobic metabolic activity. Metabolic pathway enrichment analysis (Fig. 8E) further highlighted that the metabolic disturbances induced by MPs were predominantly concentrated in glycolysis/gluconeogenesis, the TCA cycle, and the pentose phosphate pathway. These findings indicate that MPs exposure significantly disrupts cellular energy metabolism, driving cells to shift from efficient aerobic metabolism to less efficient but faster anaerobic metabolism.

### Microplastics regulate metabolic reprogramming via YAP1-Mediated crosstalk of cellular mechanics signal transduction and glycolysis

**Fig. 9:**
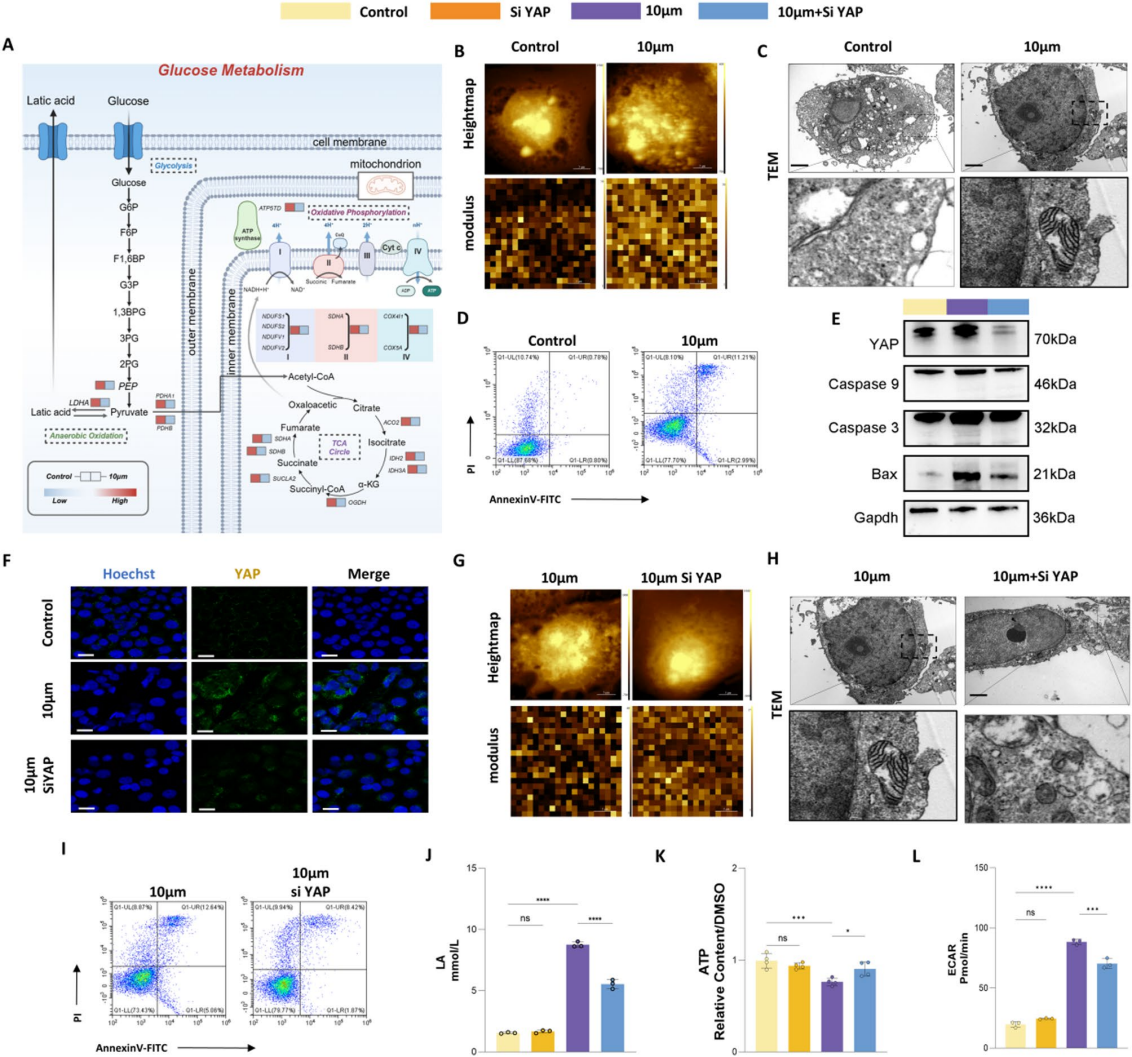
Microplastics regulate metabolic reprogramming via YAP1-mediated crosstalk of cellular mechanics signal transduction and glycolysis. (**A**) Metabolic pathway map. (**B**) Cell morphologies and moduli of Control group and 10 μm group photographed by Atomic Force Microscopy. Scale = 2 μm. (**C**) Low and high power images of cells in the Control group and 10 μm group were taken by transmission electron microscopy (TEM). Scale = 2 μm. (**D**) Cell F-Actin staining in Control group and 10 μm group. Scale = 200 μm. (**E**) Annexin V-FITC flow cytometry for apoptosis of cells in Control group and 10 μm group. (**F**) Mitochondrial apoptosis-related proteins were detected by Western Blotting. (**G**) Immunofluorescence staining of YAP protein. Scale = 200 μm. (**H**) The cell morphology and modulus of 10 μm group and 10 μm + si-YAP group were photographed by Atomic Force Microscopy. Scale = 2 μm. (**I**) Low and high power images of cells in the 10 μm group and 10 μm + si-YAP group were taken by transmission electron microscopy (TEM). Scale = 2 μm. (**J**) F-Actin staining in 10 μm group and 10 μm + si-YAP group. Scale = 200 μm. (**K**) Annexin V-FITC flow cytometry for apoptosis of cells in 10 μm group and 10 μm + si-YAP group. (**L**) Lactic acid content detection (lactic acid colorimetry). (**M**) Extracellular acidification rate detection. (**N**) ATP content detection. *p < 0.05, **p < 0.01, ***p < 0.001, ****p < 0.0001

To further investigate the metabolic reprogramming induced by large polystyrene (PS) microplastics, we integrated metabolomics and proteomics data and mapped the changes in glucose metabolism (Fig. 9A). The results revealed that multiple key proteins and metabolites involved in glucose metabolism were altered in the colon following 10 μm PS microplastic treatment. Specifically, the microplastic-treated group exhibited significant metabolic abnormalities in the glycolysis pathway. The early glycolytic metabolite fructose-1,6-bisphosphate (F1,6BP) was significantly upregulated, while late-stage glycolytic metabolites such as 2-phospho-D-glycerate (2PG) and phosphoenolpyruvic acid (PEP) were significantly downregulated, suggesting a blockade in the later stages of glycolysis. Additionally, pyruvate and lactic acid levels were significantly elevated, indicating a shift toward enhanced anaerobic metabolism to compensate for increased energy demands. Meanwhile, the accumulation of oxaloacetate in the TCA cycle, coupled with the reduction of succinate and α-ketoglutarate (α-KG), suggested a decline in TCA cycle activity. These findings imply that oxidative phosphorylation pathways were likely impaired due to mitochondrial damage, and glucose metabolism shifted toward anaerobic glycolysis, resulting in decreased ATP production.

This phenomenon indicates that MPs exposure may drive a shift in cell metabolism from aerobic to anaerobic, though the underlying molecular mechanisms remain unclear. Based on the observed changes, we hypothesized that alterations in glucose metabolism could be driven by mechanical stress at the cellular level. To test this, we validated the relationship between cellular mechanics and anaerobic metabolism. Atomic force microscopy (AFM) analysis (Fig. 9B) demonstrated a significant increase in the Young’s modulus of cells exposed to 10 μm microplastics compared to the Control group. Transmission electron microscopy (TEM) (Fig. 9C) further showed local deformations of the cell membrane and mitochondrial swelling in the 10 μm group, suggesting mechanical damage induced by MPs.

To further explore the effects of mechanical stress on the cytoskeleton, we performed phalloidin staining (Additional file6:Fig.S6). In the Control group, actin filaments appeared clear and intact, whereas in the 10 μm group, actin filaments were disordered, broken, and rearranged, indicating that mechanical damage may affect the cytoskeletal structure. We also assessed apoptosis using flow cytometry. As shown in Fig. 9D, the apoptosis rate in the Control group was 0.78%, whereas in the 10 μm group, it increased significantly to 11.2%, suggesting a marked increase in apoptosis following MPs exposure. Western blotting of mitochondrial apoptosis-related proteins (Fig. 9E) revealed no significant differences in GAPDH expression across groups. However, the expression levels of Bax, Caspase-9, and Caspase-3 were lower in the Control group and increased in the 10 μm group.

Yes-associated protein (YAP) is a well-established regulator of cell mechanics and has been implicated in glycolytic metabolism. Under physiological conditions, YAP remains phosphorylated in the cytoplasm, where it interacts with inhibitory factors, maintaining an inactive state. However, upon dephosphorylation, YAP translocates from the cytoplasm to the nucleus, a process known as nuclear translocation, which is critical for its functional activation. Given its role in cellular mechanics, this study focused on YAP involvement in damage processes induced by large-sized microplastics. Western blot analysis revealed a significant upregulation of YAP expression in the 10 μm microplastic group (Additional file 7: Fig. S7). To confirm YAP nuclear translocation, immunofluorescence staining was performed (Fig. 9F). In the Control group, YAP localization was predominantly cytoplasmic. In contrast, in the 10 μm treatment group, a marked increase in nuclear YAP-associated fluorescence was observed, indicating a significant nuclear translocation of YAP following MP exposure.

To further investigate the role of YAP in microplastic-induced mechanical damage, we knocked down YAP in the MPs treatment group (Additional file7:Fig.S7). AFM imaging (Fig. 9G) showed a significant reduction in the Young’s modulus of the 10 μm + si-YAP group compared to the 10 μm group, suggesting that microplastics regulate mechanotransduction through YAP. However, TEM (Fig. 9H) and phalloidin staining (Additional file6:Fig.S6) indicated that YAP knockdown only partially alleviated the damage to cell membrane integrity and cytoskeletal disruption, suggesting that the primary role of YAP is in signaling regulation rather than directly affecting membrane structure. Flow cytometry revealed that the apoptosis rate in the 10 μm group was 12.64%, whereas in the 10 μm + si-YAP group, it decreased to 8.62% (Fig. 9I), indicating that YAP knockdown alleviated apoptosis. Western blotting confirmed this result (Fig. 9E), showing that apoptosis-related proteins Bax, Caspase-9, and Caspase-3 were downregulated in the 10 μm + si-YAP group compared to the 10 μm group.

To explore the role of YAP in energy metabolism and metabolic reprogramming, we analyzed lactate content, extracellular acidification rate (ECAR), and ATP levels in the Control, 10 μm, si-YAP, and 10 μm + si-YAP groups (Fig. 9J-L). The results showed that lactate levels and ECAR were significantly elevated in the 10 μm group, indicating enhanced anaerobic metabolism, while ATP levels were significantly reduced, suggesting mitochondrial dysfunction and a shift toward anaerobic glycolysis. However, in the 10 μm + si-YAP group, these indicators were alleviated, with lactate and ECAR levels decreasing, and ATP levels significantly restored. These findings reveal that MPs regulate mechanosignaling via YAP, contributing to metabolic reprogramming and leading to cell apoptosis due to low ATP production. Moreover, YAP regulation can reverse the metabolic changes and alleviate cellular damage induced by MPs exposure.

### YAP inhibitor ameliorates microplastic-induced colon inflammation

**Fig. 10:**
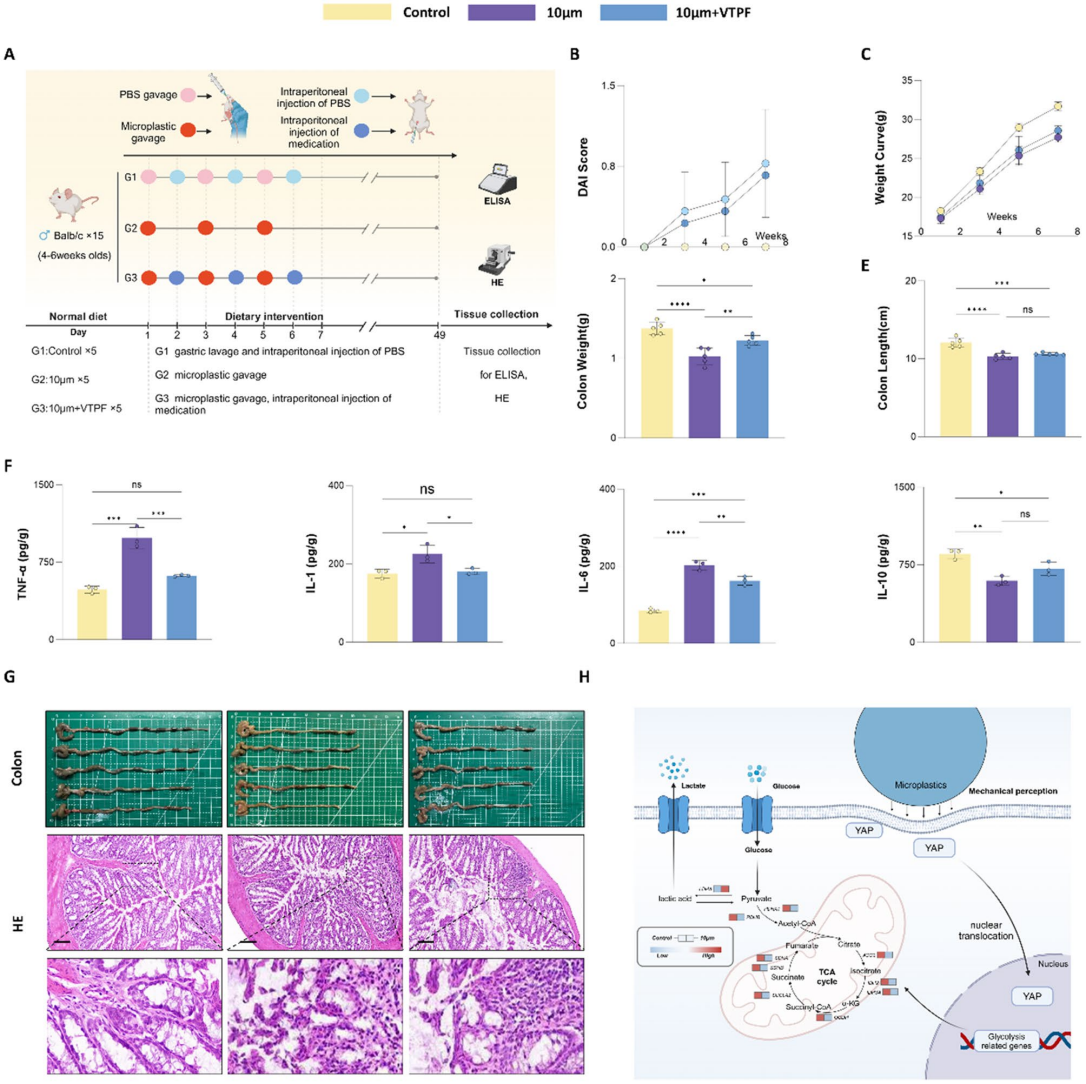
YAP inhibitor ameliorates microplastic-induced colon inflammation. (**A**) Schematic of the treatment protocol for microplastic and control groups. (**B**) DAI scores during the gavage process for microplastic and control groups. (**C**) Body weight growth curve changes during gavage in the microplastic and control groups. (**D**) Comparison of colon mass between microplastic and control groups. (**E**) Comparison of colon length between microplastic and control groups. (**F**) Expression levels of Four inflammatory factors in microplastic and control groups (ELISA). (**G**) Anatomical diagrams of the intestinal structures and H&E staining of colon tissues in the microplastic and control groups. (**H**) Schematic of mechanical sensing and metabolic reprogramming induced by 10 μm PS microplastics. *p < 0.05, **p < 0.01, ***p < 0.001, ****p < 0.0001

To explore the role of YAP in microplastic (MP)-induced colon inflammation and assess its potential as a therapeutic target, an in vivo experiment was conducted using the YAP-specific inhibitor VTPF. VTPF is a widely used YAP inhibitor that prevents YAP nuclear translocation by suppressing its activity, thereby disrupting downstream YAP-related signaling pathways. Mice were assigned to three experimental groups: Control, 10 μm microplastic exposure (10 μm group), and 10 μm microplastic exposure with VTPF treatment (10 μm + VTPF group). The 10 μm group received weekly oral gavage of microplastics, while the 10 μm + VTPF group was administered both 10 μm PS microplastics via gavage and intraperitoneal injections of VTPF. The experiment lasted for 7 weeks (Fig. 10A). Physiological parameters were continuously monitored in all groups throughout the study.

Compared to the Control group, mice exposed to 10 μm microplastics showed significant weight loss, increased Disease Activity Index (DAI) scores, shortened intestinal length, and reduced intestinal mass. In contrast, VTPF treatment exerted protective effects, evidenced by weight gain, markedly lower DAI scores, and restoration of intestinal length and mass to near-control levels (*P* < 0.05, Fig. 10B-E, Additional file 8: Fig. S8).

To further assess the anti-inflammatory effect of VTPF, we performed ELISA on intestinal tissues from euthanized mice (Fig. 10F). The results demonstrated that in the 10 μm + VTPF group, pro-inflammatory cytokine levels were significantly reduced, while the anti-inflammatory cytokine IL-10 showed partial recovery (*P* < 0.05). Additionally, histological examination of the colonic tissue via H&E staining (Fig. 10G) revealed no significant inflammation in the Control group, while typical pathological features of colitis were observed in the 10 μm group. In contrast, intestinal inflammation was significantly reduced in the 10 μm + VTPF group. These findings confirm that the YAP inhibitor VTPF can mitigate microplastic-induced intestinal inflammation, offering significant protection against microplastic-induced intestinal damage in mice.

To explore the molecular mechanisms underlying large-particle microplastic-induced intestinal damage, we constructed a mechanistic model, as depicted in Fig. 10H. Upon contact with the cell membrane, large-particle PS microplastics mechanically stimulate YAP activation, leading to nuclear translocation. The translocated YAP then regulates the expression of glycolysis-related genes, such as LDHA and PKM (validated by Western blotting, shown in the supplementary figure), promoting a shift towards glycolysis.

## Discussion

With the escalation of microplastic pollution, increasing research has focused on its impacts on ecological and human health [43]. Studies have shown that microplastics can damage intestinal epithelial cells and impair barrier integrity through oxidative stress, inflammation, and immune dysregulation [44–46]. However, microplastics span a wide size range—from nanometers to micrometers—and their toxicological mechanisms may differ accordingly. Despite this, current research has primarily addressed plastic types and exposure levels, with limited attention to particle size. Given that particle size affects uptake, immune recognition, and potentially gut microbiota composition, it likely plays a key role in intestinal toxicity. Yet, its contribution to microplastic-induced intestinal inflammation remains insufficiently understood.

This study aims to address the current knowledge gap by investigating how microplastic particle size influences the molecular mechanisms of intestinal injury. In our study, we selected 100 nm and 10 μm polystyrene particles as representative sizes for nanoplastics and microplastics, respectively. This decision was based on two key considerations: first, these sizes are among the most commonly detected in environmental samples and widely used in experimental studies, providing consistency and comparability with existing literature; second, they effectively reflect the biological differences between cellular-uptake-prone nanoparticles and mechanically disruptive microparticles. Therefore, while a broader range of particle sizes might offer additional insights, our focus on these two typical sizes was intended to highlight the distinct toxicity mechanisms associated with each class. Using both cell and animal models, we found that nano-scale polystyrene microplastics primarily induce damage through ferroptosis, whereas larger micro-scale particles act via mechanical disruption and metabolic reprogramming. Furthermore, we identified two therapeutic targets and demonstrated that intervention with Ferrostatin-1 and VTPF significantly alleviated colon injury in vivo. These findings offer new mechanistic insights and suggest potential strategies for treating microplastic-induced intestinal damage.

The core finding of this study is that microplastics of different sizes induce intestinal injury via distinct molecular pathways. Smaller particles, owing to their larger surface area, more readily penetrate the intestinal epithelial barrier [47]. Once internalized, they impair mitochondrial function and disrupt iron homeostasis, leading to oxidative stress and intracellular iron accumulation, which promotes lipid peroxidation and activates ferroptosis [48]. Ferroptosis is a regulated form of cell death driven by iron-dependent lipid peroxidation [49, 50]. The Slc7a11 system imports cystine for glutathione (GSH) synthesis, while Gpx4 reduces lipid peroxides [51–53]. GSH depletion inactivates Gpx4, causing ROS accumulation and ferroptosis initiation.

Dysregulation of the p53 tumor suppressor pathway is a hallmark of many human cancers [37, 54]. p53 plays a central role in determining cell fate, including the induction of cell cycle arrest, apoptosis, and senescence [55, 56]. Recent studies have shown that elevated ROS levels can activate p53, which in turn suppresses Slc7a11 expression and promotes ferroptosis [57]. It is plausible that nanoplastics (NPs) exacerbate intracellular oxidative stress and iron accumulation, thereby activating this pathway.

While most previous studies have focused on the ability of microplastics to induce cellular damage through oxidative stress [58–60], few have explored the potential link between microplastics and ferroptosis in colon injury. In our study, proteomic analysis revealed a significant upregulation of Fosl1 in the 100 nm exposure group. Functional assays confirmed that Fosl1 knockdown reduced ferroptosis-related proteins (Slc7a11 and Gpx4) and decreased key markers, including ROS levels, JC-1 depolarization, and MDA accumulation.

To investigate the molecular mechanism underlying Fosl1’s role in ferroptosis, we conducted ChIP-qPCR and dual-luciferase assays, which demonstrated that Fosl1 could bind to the promoter region of p53, thereby promoting p53 expression. This, in turn, downregulated the expression of SLc7a11 [22, 25, 58], further exacerbating ferroptosis. Our findings highlight the pivotal role of Fosl1 in the ferroptosis induced by NPs. This insight not only provides new molecular understanding of microplastic-induced ferroptosis but also suggests potential therapeutic targets for future treatment of microplastic-induced intestinal damage.

In vivo, flow cytometry and multiplex immunofluorescence revealed increased infiltration of dendritic cells (DCs) and CD8⁺ T cells in the colonic tissue of NP-exposed mice. In chronic inflammation, elevated DC levels often indicate immune overactivation [61]. Ferroptosis may promote antigen release, which is recognized by DCs and presented to T cells, enhancing CD8⁺ T cell recruitment. This immune-activating cascade characterizes immunogenic cell death (ICD), wherein cell death alters the immune microenvironment and triggers cytotoxic responses [62, 63].

Flow cytometry and multiplex immunofluorescence results collectively demonstrate that nanoparticles trigger a pronounced immune and inflammatory response in the intestinal microenvironment, thereby promoting immunogenic cell death. Moreover, treatment with the ferroptosis inhibitor Ferrostatin-1 [39, 64] significantly attenuated ferroptosis, which was accompanied by a marked reduction in the infiltration of activated DCs and CD8 + T cells within the tissues. These findings further support the notion that ferroptosis serves as a key initiating event in nanoparticle-induced intestinal damage. Although this study demonstrated that nanoplastics-induced ferroptosis was associated with enhanced infiltration of dendritic cells and CD8⁺ T cells, a definitive diagnosis of immunogenic cell death generally requires the assessment of specific DAMPs, such as calreticulin (CRT), HMGB1, and extracellular ATP. Although these markers were not directly measured in this study, previous reports have indicated that ferroptosis can trigger DAMP release, contributing to immune activation. Future work will focus on validating these molecular hallmarks to further confirm the ICD phenotype triggered by nanoplastics [65, 66].

Compared to nanoparticles, larger-sized microplastics are often overlooked in research due to their limited ability to be internalized by cells [67], resulting in a lack of in-depth studies on their effects. Unlike smaller nanoparticles, the damage mechanisms of larger microplastics are likely more related to physical mechanical forces or direct mechanical effects. In our study, these particles disrupted membrane integrity and cytoskeletal structure in NCM460 cells, leading to altered cell mechanics and metabolic reprogramming. Energy metabolomics combined with proteomic analysis revealed significant upregulation of glycolysis-related proteins and metabolites, indicating a metabolic shift toward anaerobic glycolysis in response to MP-induced mechanical stress.

**Fig. 11:**
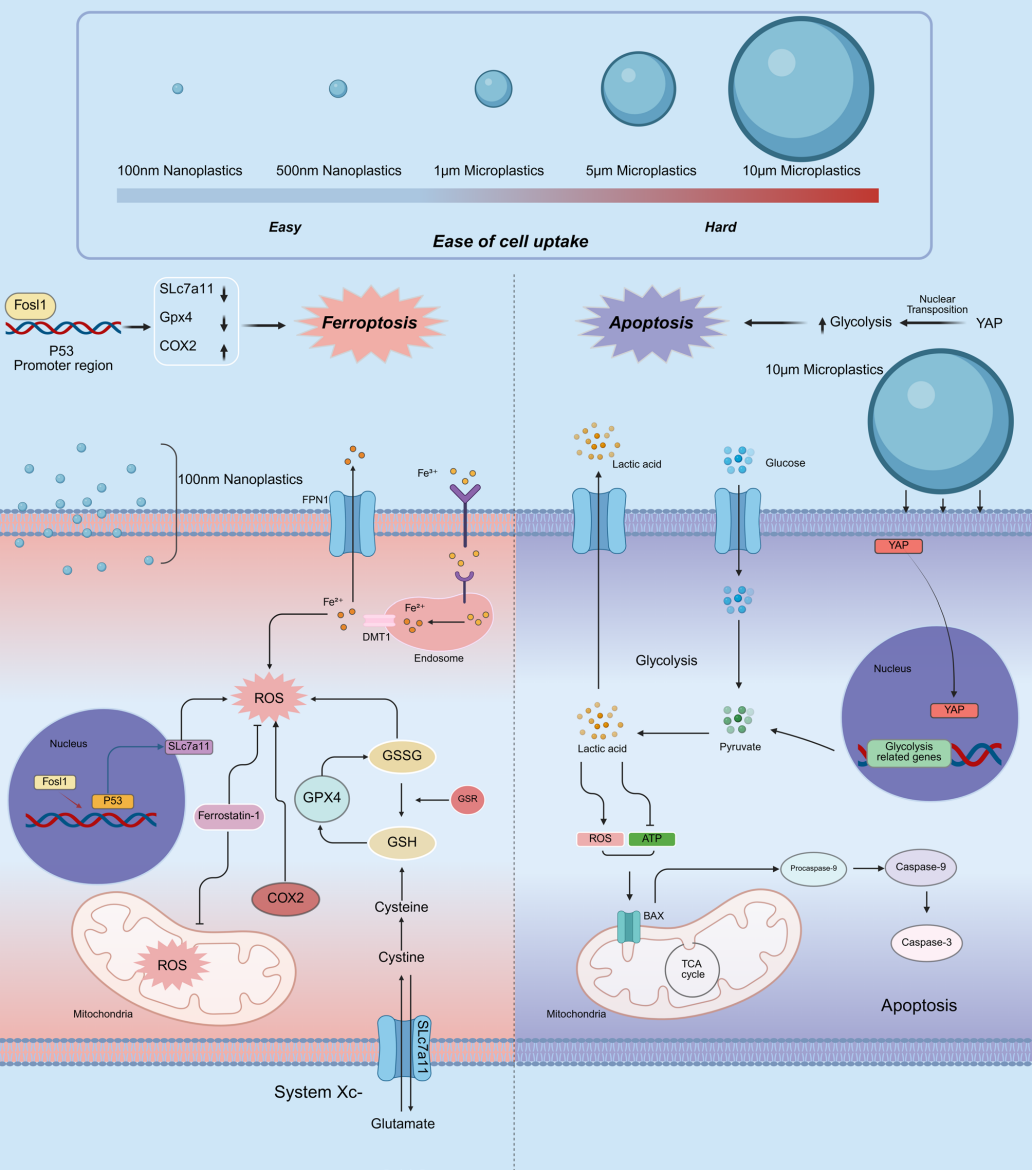
Mechanistic illustration of colonic epithelial cell damage induced by micro- and nano-sized microplastics

The metabolic disturbances caused by large microplastics appear closely linked to Yes-associated protein (YAP), a key cellular mechanosensor involved in translating mechanical stimuli into gene expression changes [68, 69]. In our study, MP exposure significantly upregulated YAP expression and induced its nuclear translocation, likely activating glycolysis-related genes. YAP is recognized as a central regulatory node connecting mechanical stress to metabolic reprogramming, particularly glucose metabolism [41, 70]. Targeting YAP offers a promising strategy to mitigate MP-induced cellular damage. Knockdown of YAP alleviated abnormal glycolytic activity, restored ATP synthesis, and reduced lactate accumulation. Furthermore, treatment with VTPF, a pharmacological YAP inhibitor, effectively attenuated intestinal injury induced by large-sized MPs. These findings highlight that microplastics can induce metabolic reprogramming through mechanical stress-mediated YAP activation and suggest that modulating this pathway may provide therapeutic benefit (Fig. 11).

Although this study reveals novel mechanisms underlying microplastic-induced intestinal damage and identifies effective therapeutic strategies, several limitations and research gaps remain. First, microplastics vary widely in chemical composition, surface properties, and environmental interactions. Most studies, including ours, focus on a single type (e.g., polystyrene), potentially overlooking the distinct or synergistic effects of other polymers such as polyethylene. Future research should systematically compare different microplastic types, exposure levels, and their combined effects with other environmental pollutants on intestinal health.

Furthermore, the influence of microplastics on the gut microbiome remains insufficiently investigated. The gut microbiome plays a critical role in maintaining intestinal health and immune function [71, 72]. Microplastic exposure may disrupt the structure of the gut microbiome, which could subsequently affect gut barrier function, immune responses, and metabolism [73, 74]. Future studies could integrate microbiome analysis with metabolomics technologies to investigate the effects of microplastic exposure on the gut microbiome and its potential role in intestinal damage.

The impact of microplastics on gut health is not just a biological issue but also a growing public health concern [43, 75, 76]. As environmental exposure increases, microplastics have been detected in humans via multiple routes—including the food chain, drinking water, and airborne particles—with the gastrointestinal tract as a major site of accumulation [77, 78]. Microplastics may contribute to the development of multiple chronic diseases, including intestinal inflammation and metabolic disorders [79]. Although this study proposes promising therapeutic strategies for microplastic-induced gut injury, their clinical translation requires further validation through extensive preclinical and clinical studies.

Although no unified international standard currently exists for human microplastic exposure, previous studies estimate that humans may ingest approximately 0.1–5 g of microplastics per week, depending on regional environmental levels and dietary habits. Based on body surface area conversion, this corresponds to an approximate equivalent exposure dose of 0.054–2.71 mg/day in mice [74]. The doses used in this study fall within or slightly above this range, particularly modeling a high-exposure scenario that may be encountered in contaminated environments. Therefore, the exposure concentrations we selected remain biologically relevant and offer valuable insights into the potential health risks associated with heavy microplastic burdens.

Moreover, our pre-experiments showed that concentrations below 1 mg/mL did not significantly affect cell viability, indicating that these doses may fall below the biological threshold for measurable toxicity in vitro. This supports our decision to use 1 mg/mL as the lower bound in mechanistic studies. Nevertheless, we fully acknowledge the importance of exploring long-term, low-dose exposure to better mimic chronic human exposure. Future studies will further investigate the sub-toxic effects of nanoplastics at environmentally relevant concentrations over extended exposure periods.

## Conclusion

This study comprehensively elucidates the size-dependent mechanisms by which polystyrene (PS) MPs induce intestinal epithelial damage. Our findings demonstrate two distinct and non-overlapping toxicological pathways: small-sized MPs (100 nm) trigger ferroptosis-driven immune injury, while large-sized MPs (10 μm) induce inflammation through mechanical disruption and metabolic reprogramming.

Specifically, 100 nm MPs were internalized by intestinal epithelial cells via endocytosis, leading to intracellular oxidative stress and ferroptosis. Proteomic analysis revealed a significant upregulation of Fosl1 and enrichment in the ferroptosis and p53 pathways. Ferrostatin-1, a ferroptosis inhibitor, significantly alleviated both cellular and tissue damage, confirming ferroptosis as a central mechanism in small MP-induced intestinal toxicity. Additionally, Fosl1 knockdown reduced ferroptosis-related damage, further highlighting its regulatory role.

In contrast, 10 μm MPs did not enter cells but caused epithelial injury via physical compression, cytoskeletal disruption, and metabolic reprogramming. Notably, these large particles activated the YAP signaling pathway, which promoted glycolysis and intensified inflammation. Inhibition of YAP by Verteporfin (VTPF) significantly reversed these effects, validating YAP as a key mediator of microplastic-induced intestinal injury in larger particles.

Together, these results uncover critical particle size-specific mechanisms—ferroptosis for nanoscale MPs and YAP-mediated glycolytic reprogramming for microscale MPs. These mechanistic distinctions offer novel insight into microplastic toxicity, establish Fosl1 and YAP as potential therapeutic targets, and provide compelling experimental evidence for tailored intervention strategies against microplastic-induced gut damage.

In conclusion, this study not only advances our molecular understanding of how MPs of different sizes affect intestinal health, but also delivers important theoretical support for microplastic risk assessment, environmental regulation, and public health protection.

## Electronic supplementary material

Below is the link to the electronic supplementary material.

Supplementary Material 1

Supplementary Material 2

## Data Availability

The data are available to academic researchers upon request. Raw data generated and/or analysed during the current study are available from the corresponding author, upon reasonable request.
