# Supplementary material for: Molecular mechanism differences between nanoplastics and microplastics in colon toxicity: nanoplastics induce ferroptosis-mediated immunogenic cell death, while microplastics cause cell metabolic reprogramming
Source: J Nanobiotechnology. 2025 Jul 14;23:505. doi: 10.1186/s12951-025-03545-1 (PMC12261845; doi:10.1186/s12951-025-03545-1)
Supplement: Supplementary file 1 — Supplementary Material 1 [file 12951_2025_3545_MOESM1_ESM.docx]

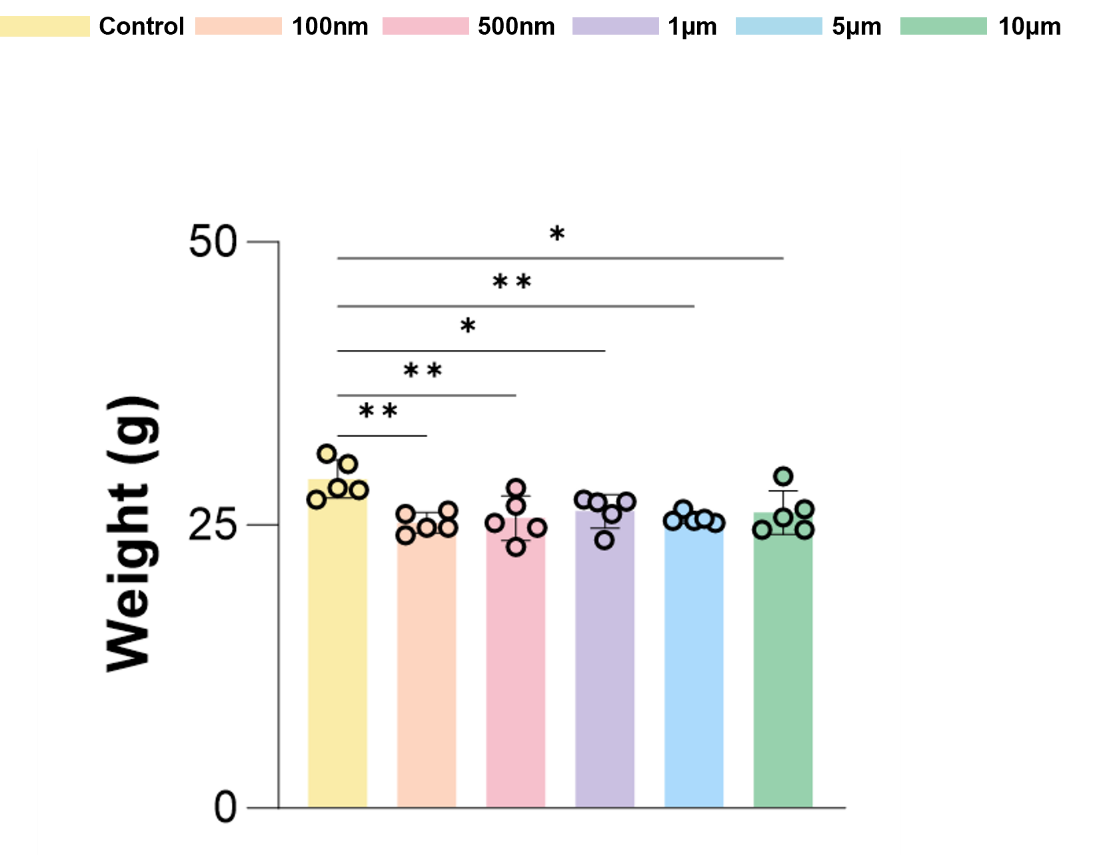


Figure S1:Comparison of body weight between microplastic group and control group (Control,100 nm, 500 nm, 1 μm, 5 μm, and 10 μm).


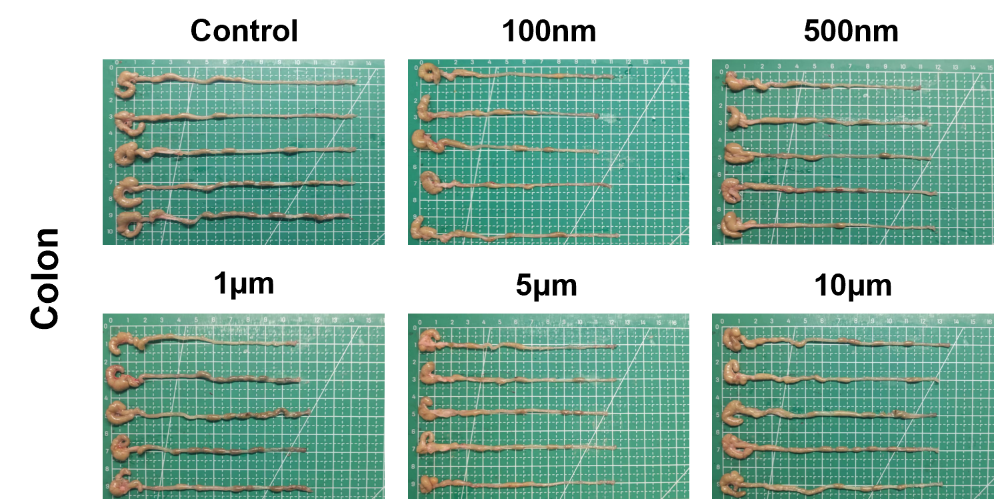


Figure S2:The microplastic group and the control group took intestinal pictures(Control,100 nm, 500 nm, 1 μm, 5 μm, and 10 μm).


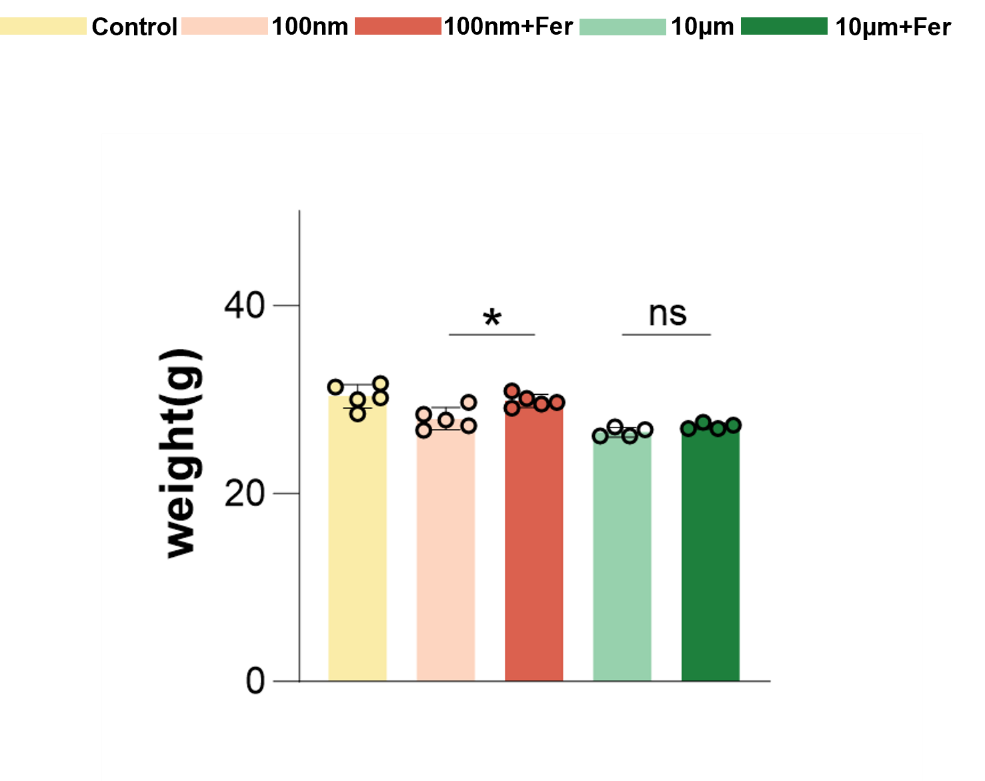


Figure S3:Comparison of body weight between microplastic group and control group (Control,100nm,100nm+Fer,10μm,10μm+Fer).


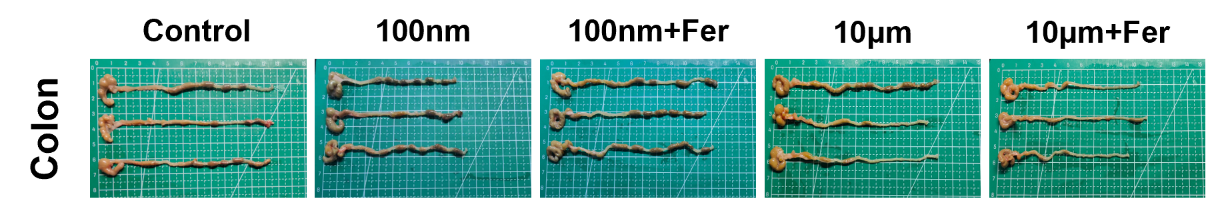


Figure S4:The microplastic group and the control group took intestinal (Control,100nm,100nm+Fer,10μm,10μm+Fer).


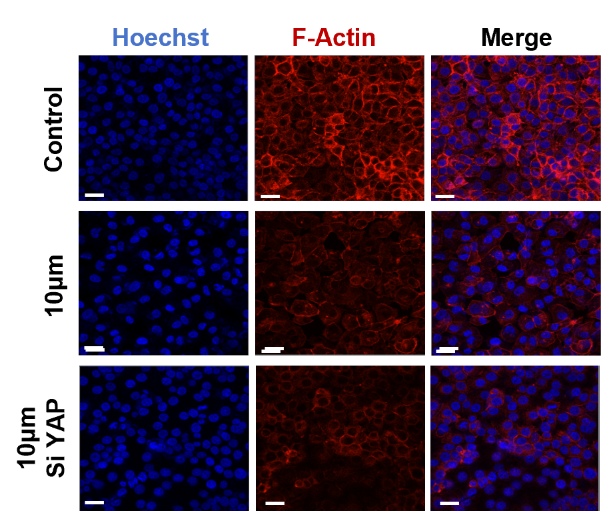


Figure S5:phalloidin staining(Control,10μm,10μm+SiYAP).


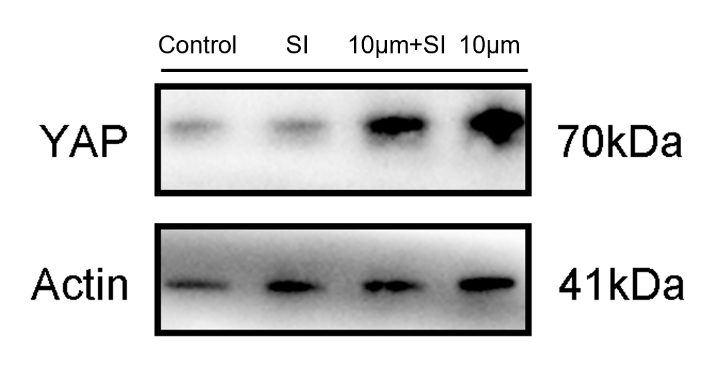


Figure S6:YAP proteins were detected by Western Blotting.


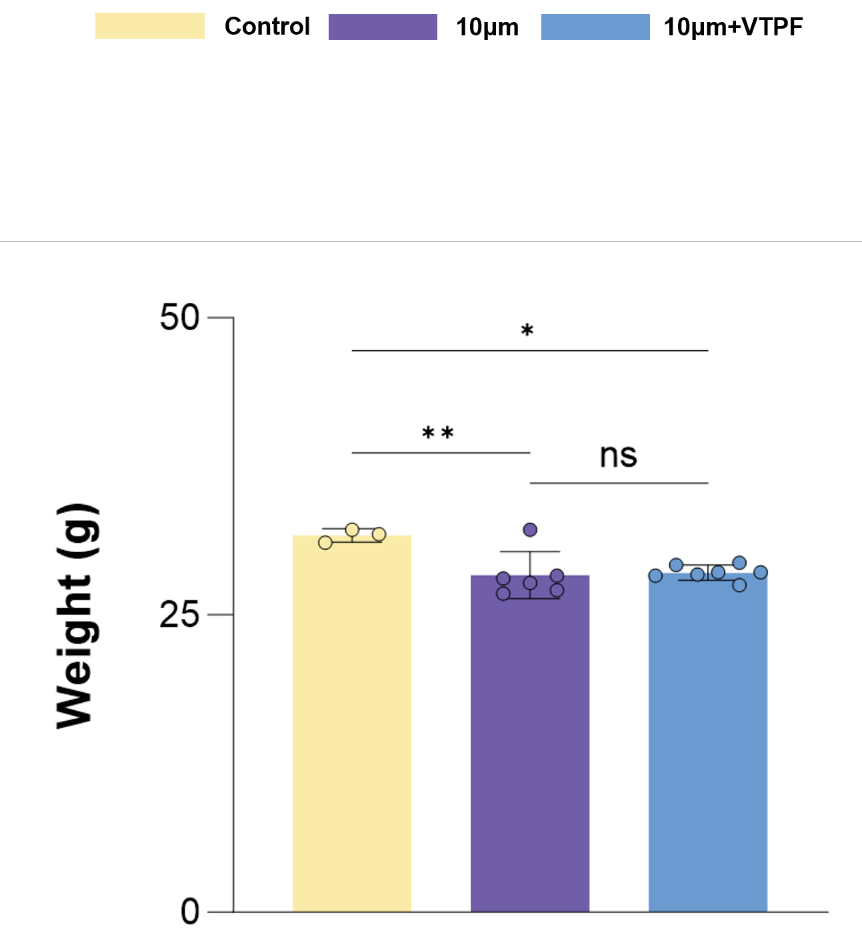


Figure S7:Comparison of body weight between microplastic group and control group (Control,10μm,10μm+VTPF).


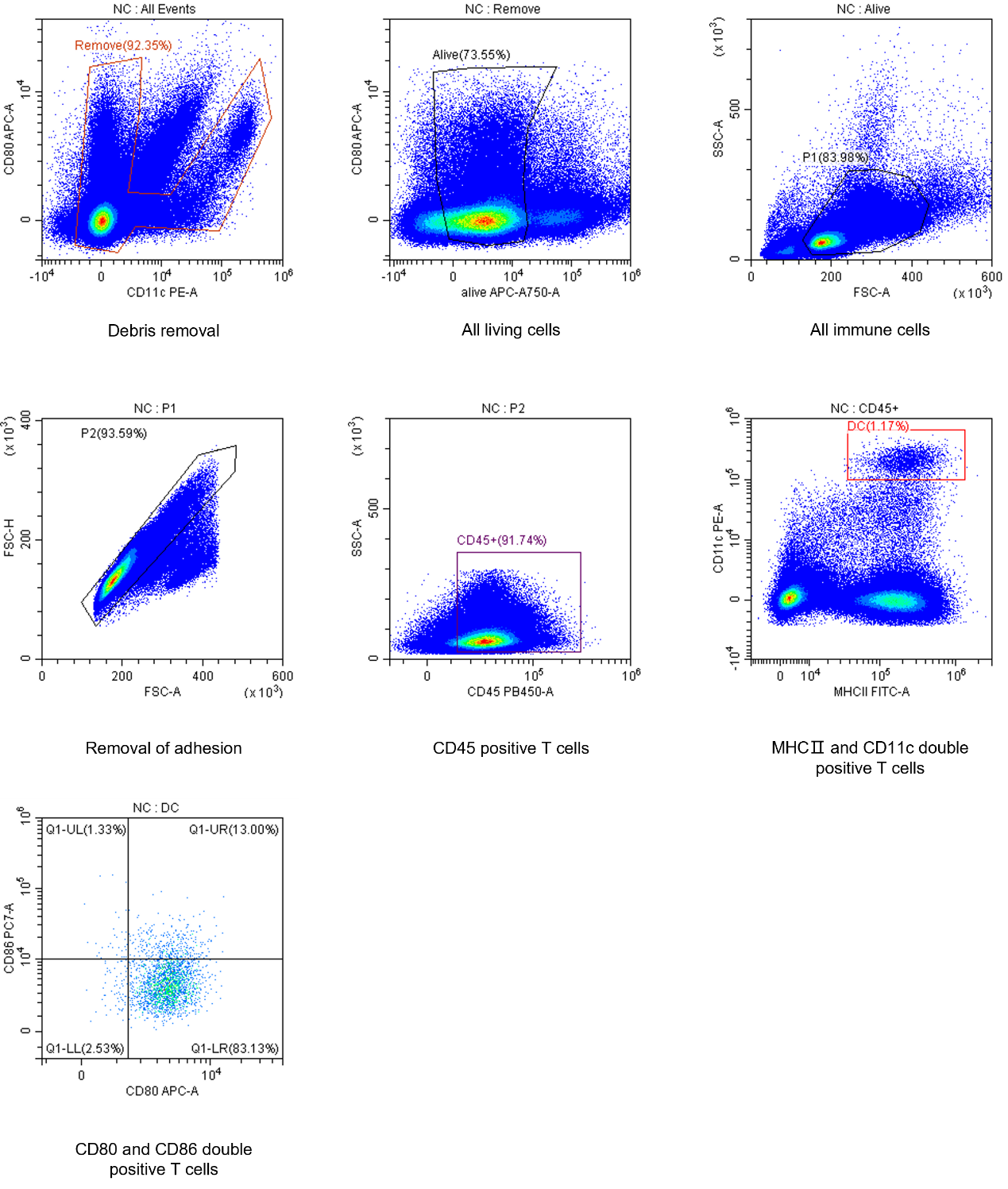


Figure S8:DC cell immunoflow Flow cytometry gate.


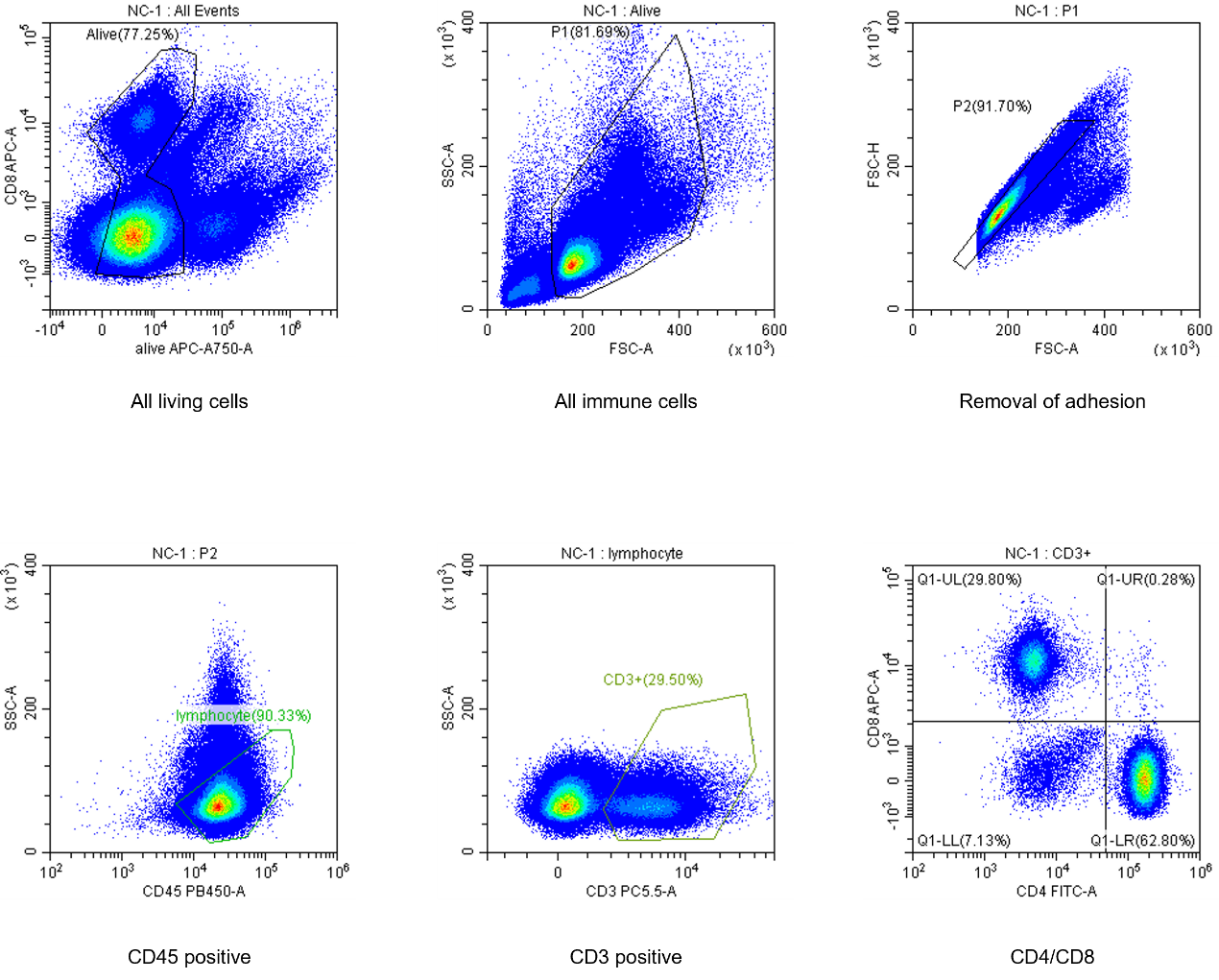


Figure S9:CD4/CD8 ratio immunoflow Flow cytometry gate.
